# Public Health Responses to and Challenges for the Control of Dengue Transmission in High-Income Countries: Four Case Studies

**DOI:** 10.1371/journal.pntd.0004943

**Published:** 2016-09-19

**Authors:** Elvina Viennet, Scott A. Ritchie, Craig R. Williams, Helen M. Faddy, David Harley

**Affiliations:** 1 Research School of Population Health, The Australian National University, Canberra, Australian Capital Territory, Australia; 2 Research and Development, Australian Red Cross Blood Service, Kelvin Grove, Queensland, Australia; 3 School of Public Health, Tropical Medicine and Rehabilitation Sciences, James Cook University, Cairns, Queensland, Australia; 4 Sansom Institute for Health Research, University of South Australia, Adelaide, SA, Australia; University of Rhode Island, UNITED STATES

## Abstract

Dengue has a negative impact in low- and lower middle-income countries, but also affects upper middle- and high-income countries. Despite the efforts at controlling this disease, it is unclear why dengue remains an issue in affluent countries. A better understanding of dengue epidemiology and its burden, and those of chikungunya virus and Zika virus which share vectors with dengue, is required to prevent the emergence of these diseases in high-income countries in the future. The purpose of this review was to assess the relative burden of dengue in four high-income countries and to appraise the similarities and differences in dengue transmission. We searched PubMed, ISI Web of Science, and Google Scholar using specific keywords for articles published up to 05 May 2016. We found that outbreaks rarely occur where only *Aedes albopictus* is present. The main similarities between countries uncovered by our review are the proximity to dengue-endemic countries, the presence of a competent mosquito vector, a largely nonimmune population, and a lack of citizens’ engagement in control of mosquito breeding. We identified important epidemiological and environmental issues including the increase of local transmission despite control efforts, population growth, difficulty locating larval sites, and increased human mobility from neighboring endemic countries. Budget cuts in health and lack of practical vaccines contribute to an increased risk. To be successful, dengue-control programs for high-income countries must consider the epidemiology of dengue in other countries and use this information to minimize virus importation, improve the control of the cryptic larval habitat, and engage the community in reducing vector breeding. Finally, the presence of a communicable disease center is critical for managing and reducing future disease risks.

## Introduction

Pathogens and vectors can now be transported rapidly around the world. Consequently, new infections are emerging and some old infections, including dengue, have re-emerged. Dengue virus (DENV), a flavivirus, is transmitted by *Aedes* mosquitoes and causes large epidemics and endemic transmission in nonimmune populations, particularly in economically disadvantaged tropical and subtropical countries. Some high-income countries (HICs) or states such as Aruba, Brunei Darussalam, Puerto Rico, and Singapore are dengue-endemic. Others, such as the state of Queensland in Australia, French Polynesia, New Caledonia, and Taiwan experience regular dengue outbreaks, whereas still others, such as The Bahamas, Bermuda, France, Japan, and the state of Florida in the United States, experience small outbreaks.

The incidence of dengue has grown markedly throughout the world in recent decades. Dengue is under-reported, but the actual number of dengue infections per year is estimated to be as high as 390 million (95% credible interval, 284–528 million), of which 96 million (67–136 million) manifest clinically [1]. According to the World Health Organization [2], in 2012 the number of deaths caused by dengue was 297, 873, and 159 per million in low-income, lower middle-income and upper middle-income countries, respectively. The number of deaths is lower (38 per million) in HICs [2].

**Fig 1:**
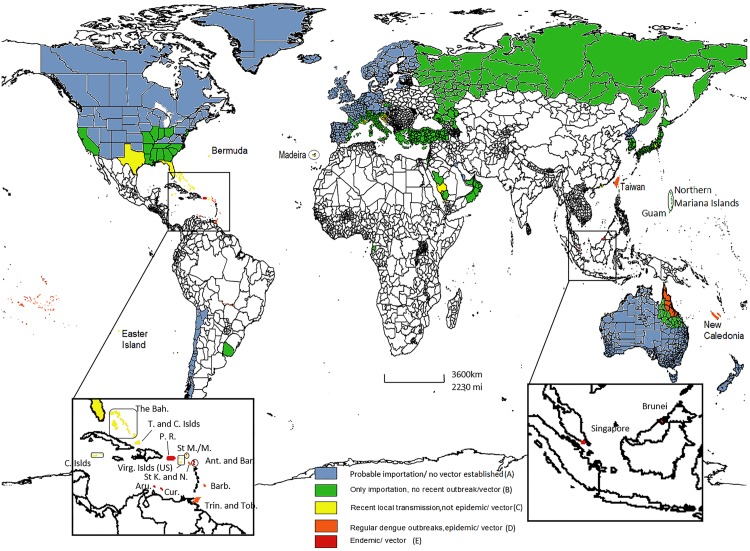
Occurrence of dengue in HICs as of December 2015.

The World Bank Atlas method is used to calculate gross national income (GNI). GNI is a useful and easily available indicator that correlates closely with other, nonmonetary measures of quality of life, such as life expectancy at birth, mortality rates for children, and enrollment rates in school. Economies are defined according to the GNI per capita in 2013 as: low income, US$1,045 or less [3]; middle income, more than US$1,045 but less than US$12,746; and high income, US$12,746 or more [3]. The dengue vector is established in 47 of the 76 HICs listed by the World Bank, and 29 have neither a dengue vector nor local DENV transmission. Nineteen of the 47 HICs have reported only imported cases of dengue, and the other 28 countries have reported recent autochthonous transmission (S1 Table). Of these 28 HICs with local transmission, 14 have reported only small numbers of cases (including Florida), eight have regular large outbreaks (including Queensland and Taiwan), and six are classified as dengue endemic (including Singapore) (Fig 1 and S1 Table).

Dengue poses an economic burden, especially in resource-poor, dengue-endemic countries [4,5]. Despite its lower prevalence in HICs, dengue also has economic costs in these countries [6,7]. Costs per case are generally higher in HICs such as Argentina, Australia, Brunei Darussalam, Singapore, and the US [8]. It is unclear why dengue is transmitted despite intensive surveillance and control programs in HICs [9,10]. The aim of this review is to discuss the relative burden of dengue in four representative HICs. We describe the unique features of dengue and provide a detailed review of the main drivers of dengue transmission in these HICs. We then discuss the public health responses and challenges for public health programs, and we finish with some recommendations for the future.

We restricted our analysis to Singapore, Taiwan, Australia, and the US, with a particular focus on the states of Queensland in Australia and Florida in the US. Queensland and Florida have many similarities [11]. Both have similar weather patterns (tropical to temperate) [12,13] and have experienced population growth because of their favorable climate [11]. East Central Australia and East Central Florida have natural features of ecological significance for mosquito habitats [14]. The environment and ecology of Queensland and Florida are fairly similar and comprise coastal ecosystems, rainforest, freshwater wetlands, floodplain forests, and woodlands [15,16]. Both states contain areas where *Aedes* (*Stegomyia*) *aegypti* (L.), the principal vector of dengue, chikungunya and Zika viruses, is established. Singapore and Taiwan are dengue-endemic and -nonendemic countries, respectively, and are examples of countries with increasing rates of autochthonous dengue and dengue hemorrhagic fever (DHF) despite intensive vector control programs. Lessons learned within these countries provide valuable public health and epidemiological information for other high-income nonendemic countries at risk for the establishment of dengue.

## Literature Search

To review this topic, we searched PubMed, ISI Web of Science, and Google Scholar for articles published up to 05 May 2016. The following terms were used: “dengue fever,” “dengue,” “outbreaks,” “transmission,” “burden,” “high-income countries,” and “World Bank income countries” in combination with the name of each of the 76 HICs. Relevant articles referenced in publications were identified by searching the lead author’s personal files in Google Scholar and in the Literature Retrieval System of the Armed Forces Pest Management Board, US. Articles in English, Spanish, and French were reviewed. ProMED-mail [17], Eurosurveillance [18], and DengueMap [19] websites were used to complete the search.

## Relative Burden of Dengue in Selected HICs

The number of countries that experienced severe dengue epidemics increased from nine before 1970 to more than 100 in 2015 [20]. DENV now occurs in regions of Africa, the Americas, the eastern Mediterranean, southeast Asia, and the western Pacific [20].

Notwithstanding differences in reporting methods, the economic impacts of dengue on a variety of measures are considerable for HICs. These impacts can be direct, such as the costs of treatment, hospitalization, and prevention, or indirect, such as the loss of productivity related to absence, disability, or death and the effects on tourism [21]. Table 1 presents epidemiological data for dengue infection in Queensland (Australia), Florida (US), Singapore, and Taiwan.

**Table 1:** The burden of dengue fever, highlighting the number of locally acquired cases in Queensland (Australia), Florida (US), Singapore, and Taiwan.

	Years, location	Serotype(s)*	N dengue cases	N locally acquired cases	N hospitalizations (%)	Incidence per 100,000	N DHF (%)	N deaths (Case Fatality Rate %)	Ref
**Queensland, Australia**	1996–1997, the Torres Strait	DENV-2	208	202	7 (3.48)	6.49	0	0	[22,23]
	1997–1999, Cairns; Port Douglas; Mossman	DENV-3	498	493	101 (20.28)	14.86	6 (1.20)	0	[24–26]
	2000, Cairns	DENV-2	85	50	0	2.44	0	0	[25,27]
	2001, Townsville	DENV-2	42	8	1 (11.11)	1.17	4 (9.52)	0	[27]
	2002, Kuranda; Townsville; Cairns	DENV-2; -1; -4	81	25	0	2.22	0	0	[27,28]
	2003–2004, Cairns; Townsville; the Torres Strait	DENV-2	999	895	35 (3.91)	26.34	4 (0.45)	2 (0.20)	[27,29,30]
	2005, the Torres Strait; Townsville	DENV-4	116	76	0	3.04	0	0	[27,31]
	2006, Townsville; Cairns	DENV-3; -2	76	37	0	1.95	0	0	[27,31]
	2007, Townsville	DENV-3	119	47	0	2.93	0	0	[27,31]
	2008–2009, Port Douglas; Mossman; Townsville; Cairns	DENV-3; -3; -3, -1; -2, **-3**	1,314	1,042	73 (5.56)	26.58	6 (0.46)	1 (0.11)	[26,27,31,32]
	2010–2011, Cairns; Townsville	DENV-1, -2, -3, -4; -1, -2, -3	474	148	-	10.94	0	0	[31,33]
	2012, Cairns; Townsville	**DENV-1**, -2; -1, -2	244	28	-	5.30	0	0	[31]
	2013, Port Douglas, Mossman; Cairns	DENV-3; -1	490	222	-	10.45	0	0	[31,34]
	2014, Cairns; Innisfail; Port Douglas; Charters Towers; Townsville	DENV-1; -1; -3	395	182	-	8.32	0	0	[31,34]
	2015, Cairns, Tully, Innisfail, El Arish, Townsville	DENV-1; -2	284	62	-	-	0	0	[35,36]
**Florida**	2009, Key West	DENV-1	63	27	0	0.3	0	0	[37,38]
	2010–2011, Key West; Broward; Miami-Dade Counties	DENV-1, -2	257	65	0	1	0	0	[37–42]
	2011, Miami-Dade, Martin, Hillsborough, Palm Beach Counties	DENV-1, -2	68	7	-	-	-	-	[43,44]
	2012, Miami-Dade, Seminale, Osceola Counties	DENV-1, -4, -2, -3	139	3	0	0.7	0	0	[39,41,45]
	2013, Martin, Miami-Dade Counties	DENV-1, -4, -3, -2	149	23	6 (3.87)	0.7	0	0	[39,41,46,47]
	2014, Miami-Dade County	DENV-1, -2, -3, -4	51	6	0	0.2	0	0	[39,41,48]
	2015, Broward County	DENV-1, -2	85	1	-	-	-	-	[49]
**Singapore**	2000, Tampines regional centre, Geylang, Marine Parade	**DENV-1, -2**, -4, -3	673	402	572 (0.85)	9.3	10 (1.5)	2 (0.30)	[50,51]
	2001, Toa Payoh, Kallang, Hougang, Yishun, Novena	**DENV-2,** -4, -1	2,372	2,064	1,850 (0.78)	46.7	6 (0.30)	4 (0.17)	[51,52]
	2002, Bukit Batok; Geylang; Novena; Sengkang, Jurong Ouest; Woodlands	**DENV-2**, -1, -3, -4	3,945	3,560	3,156 (0.80)	86.2	8 (0.20)	4 (0.10)	[53,54]
	2003, Ang Mo Kio; Bedok; Hougang; Novena	**DENV-2,** -1, -3, -4	4,788	4,542	3,734 (0.78)	108.5	-	6 (0.13)	[53]
	2004, Yishun; Ang Mo Kio; Geylang; Bedok	**DENV-1**, -2, -3, -4	9,459	9,297	7,716 (0.81)	223.1	168 (1.78)	9 (0.10)	[9,53]
	2005, Yishun; Woodlands; Kallang; Toa Payoh; Jurong; Bukit Batok	**DENV-1**, -2, -3, -4	14,006	13,818	10,504 (75)	333.1	381 (2.72)	27 (0.19)	[9,55]
	2006, Bukit Batok, Woodlands, Ang Mo Kio, Pasir Ris, Clementi	**DENV-1**, -2, -3, -4	3,127	2,844	-	71.0	75 (2.40)	10 (0.32)	[51,56]
	2007, Bukit Batok; Pasir Ris; Woodlands	**DENV-2**, -1, -3, -4	8,826	8,637	-	192.3	189 (2.14)	24 (0.27)	[57,58]
	2008, Hougang, Jurong Ouest, Serangoon, Woodlands, Ang Mo Kio	**DENV-2**, -1, -3, -4	7,031	6,631	-	145.3	84 (1.19)	10 (0.14)	[59–61]
	2009, Clementi, Bukit Merah, Hougang, Kallang	**DENV-2**, -1, -3, -4	4,497	4,187	-	90.2	46 (1.02)	8 (0.18)	[59,60,62]
	2010, Woodlands, Telok Blangah, Newton, Pasir Ris	**DENV-2**, -1, -3, -4	5,363	4,978	-	105.6	34 (0.63)	6 (0.11)	[59,60,63]
	2011, Paya Lebar, Woodlands, Serangoon, Yishun, Pasir Ris, Tampines	**DENV-2**, -1, -3, -4	5,330	5,099	-	102.8	22 (0.41)	6 (0.11)	[59,60,64]
	2012, Ang Mo Kio, Bedok, Woodlands, Clementi, Hougang	**DENV-2**, -1, -3, -4	4,632	4,369	-	87.2	30 (0.64)	2 (0.04)	[60,65]
	2013, Tampines, Yio Chu Kang; Serangoon area, Bedok, Pasir Ris, Jurong, Choa Chu Kang	**DENV-1**, -2, -3, -4	22,170	21,863	-	410.6	93 (0.42)	8 (0.04)	[59,60]
	2014, Chuan Drive; Bukit Panjang, Choa Chu Kang, Hougang, Bukit Timah, Bedok, Sengkang	**DENV-1**, -2, -3, -4	18,326	17,812	3,665 (20.00)	335.0	20 (0.11)	6 (0.03)	[66–68]
	2015, Ang Mo Kio, Bishan North	**DENV-2**	11,298	-	2259 (20.00)	-	12 (0.11)	4 (0.04)	[67,69]
**Taiwan**	2000–2001, Tainan City, Kaohsiung City	DENV-2, -4	418	340	-	0.6	-	-	[70,71]
	2002, Kaohsiung City and Kaohsiung County; Pingtung County	DENV-2	5,388	5,336	-	23.9	241 (4.50)	21 (0.39)	[70,72]
	2003, Kaohsiung City	DENV-2	145	86	-	0.6	2 (23.25)	1 (1.16)	[70,72]
	2004–2005, Pingtung City, Kaohsiung City, Kaohsiung City	DENV-1, -3	733	538	-	1.8	8 (1.49)	0	[70,72]
	2006, Kaohsiung City; Kaohsiung County; Tainan City; Tainan County; Pingtung County	**DENV-3**, -1, -2	1,074	965	-	4.7	19 (1.97)	5 (0.47)	[70,72]
	2007, Tainan City; Kaohsiung City	DENV-1	2,179	2,000	-	9.5	11 (0.55)	1 (0.05)	[70,72]
	2008, Kaohsiung City; Kaohsiung County	DENV-1	714	488	-	3.1	-	-	[45,73]
	2009, Kaohsiung City; Kaohsiung County; Pingtung County	**DENV-3**	1,052	848	-	4.5	-	-	[45,73]
	2010, Kaohsiung City; Taipei City; Tainan City; Tainan County	-	1,896	1,592	-	8.1	-	2 (0.11)	[73]
	2011, Kaohsiung City; Pingtung County; Taipei; Penghu county	-	1,702	1,545	-	7.3	18 (1.05)	5 (0.29)	[70]
	2012, Tainan City; Kaohsiung City	**DENV-2**	1,478	1,271	-	6.3	20 (1.35)	5 (0.34)	[73,74]
	2013, Tainan City; Pingtung County; Taipei; Greater Kaohsiung; New Taipei City	**DENV-1**	860	596	-	3.6	14 (2.35)	1 (0.17)	[70]
	2014, Kaoshiung City; Chiayi City; Pingtung City	**DENV-1**	15,732	15,492	-	66.2	136 (0.88)	20 (0.12)	[75,76]
	2015, Tainan City; Kaoshiung City; Pingtung City	-	42,916	42,572	>44 (0.10)	-	-	209 (0.49)	[77]

* serotype known by place is separated by “;”. Dominant serotype, when known, is given in bold font.

### Epidemiological Burden of Dengue

#### Queensland, Australia

DENV is the third most common mosquito-borne virus infecting Australians after the alphaviruses Ross River and Barmah Forest viruses. The first reference to dengue in Australia appeared in *The Australian Medical Journal* in 1873 and highlighted the importation of eight cases from Mauritius via the ship *Charles Auguste* [25]. From 1896–1897, in Charters Towers in North Queensland, Hare published the first reports of dengue fever based on clinical data collected from a number of practitioners in Queensland and observed that comorbidity (diabetes, alcoholism) contributed to the severity of dengue in old patients [78]. Since the early 1990s, DENV outbreaks have increased in severity, frequency, and length in northeast Australia despite increased public health control efforts [22,79].

The risk of local transmission is currently restricted to northeast of Queensland (specifically, the northern half) where *Ae*. *aegypti* is present and where viremic travelers arriving from dengue-endemic, mainly low-income, countries in the Asian-Pacific region regularly introduce the virus [22,80,81].

**Fig 2:**
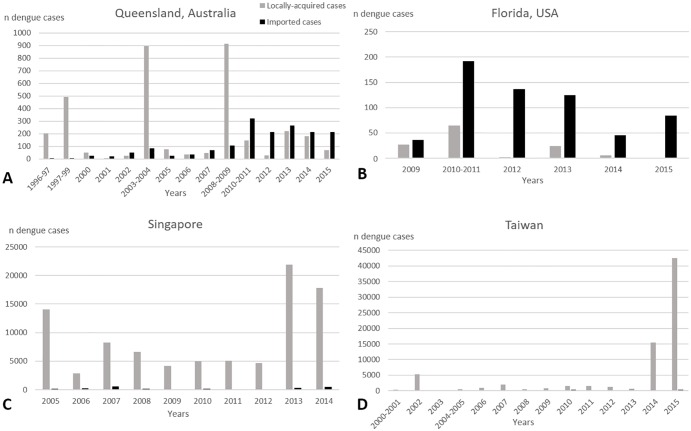
Number of locally acquired and imported dengue cases.

Imported cases are reported throughout Australia, and the number and frequency of overseas-acquired dengue cases is increasing [33,82,83] (Fig 2A). The largest number of locally acquired dengue cases (over 1,000 cases) recorded in Queensland occurred from 2008–2009 [32]. Severe DHF and dengue shock syndrome are rare in North Queensland [84]. There have been 20 DHF cases since 1996 in Australia, and three fatalities related to dengue fever nationwide in more than a century [32].

#### Florida, US

Dengue is now the leading cause of acute febrile illness in US travelers returning from the Caribbean, South America, and Asia [85]. Early reports of dengue in Florida include outbreaks in the Dry Tortugas in 1903 and Miami in 1904 and 1908 [86]. Outbreaks of dengue were reported later in 1922 and 1934 in Florida [87]. Since 1934 in Florida, and since 1945 in the rest of continental US (except for the Texas–Mexico border), no autochthonous dengue cases were reported until 2009. However, in 2009–2010, an outbreak of dengue was identified in Key West, southern Florida, where there were 22 cases of locally acquired dengue in the summer and autumn of 2009 and 66 cases in 2010. No dengue cases have been reported in Key West since November 2010 [88]. Between November 2010 and December 2014, there were 2,463 cases for the entire continental US; the two states reporting most of the imported dengue cases were New York (*n* = 574) and Florida (*n* = 495) [40]. Florida reported the largest number of locally acquired dengue cases (*n* = 97) with peaks in 2010 (60%) and 2013 (25%) [41] (Fig 2B). In 2011 and 2012, only isolated cases of autochthonous DENV transmission were reported in Florida, and none were reported in Key West. In mid-August 2013, local transmission was reported from Martin County in east-central Florida. By late September, a total of 22 locally acquired dengue cases were reported from Rio and nearby Jensen Beach [89]. An additional dengue case was reported in 2013 from Miami-Dade County. During the outbreak in 2013, which involved the DENV-1 serotype, 24 of 28 cases were symptomatic, and six of the 24 patients were hospitalized [90]. No DHF- or dengue-associated deaths have been reported in Florida.

#### Singapore

Singapore is hyperendemic for dengue, and all four serotypes (DENV-1 to -4) circulate [91]. The number of dengue cases has surged since the 1990s. Until 2013, epidemic cycles were occurring every 5–6 years with peaks in July and August. Infection and mortality in Singapore are reported principally among adults [92]. A retrospective case-control study was performed for the period 01 January to 31 September 2004 and included all cases of dengue-associated mortality treated in Tan Tock Seng Hospital, Singapore [93]. Of the seven patients who died, five (71.4%) were male [93]. In 2005, 14,006 cases were reported, and about 80% were among adults [9]. After small numbers of cases in 2006, Singapore experienced another large DENV outbreak in 2007, with 8,826 cases [94] (Fig 2C). That same year, the serotype DENV-2 re-emerged with a clade replacement and overtook DENV-1, which had been circulating since 2004, and remained the predominant serotype until 2013 [57]. A change in the predominant DENV serotype may have triggered a spike in dengue cases because of a lack of serotype-specific immunity [9]. In 2013, there was a record 22,248 reported dengue cases, with 842 dengue cases in a single week [95]. The risk of acquired dengue for nonimmune visitors ranged from 42/100,000 during a low season of a nonepidemic year to 1,700/100,000 during the high season of an epidemic year in Singapore [96].

#### Taiwan

In Taiwan, several DENV outbreaks occurred before 1945, and then there were no outbreaks until 1981 [97]. Before 2014, the largest and most severe outbreak happened in 2002 (5,388 and 241 DHF cases) [72]. In 2002, there were fewer imported cases in the southern (n = 13) compared with the northern (*n* = 28) parts of Taiwan probably because of fewer visits from travelers and fewer workers from endemic countries in the south. However, 98% of locally acquired cases occurred in the south where *Ae*. *aegypti* is most abundant [98]. Since 2011, the Centers of Disease Control, ROC (Taiwan), has reported more than 800 imported cases and 18,500 locally acquired dengue cases, with a significant increase in 2014 [99,100] (Fig 2D). For the first time since formal records were first kept in 1981, the largest DENV outbreak occurred in 2015 with 42,916 reported cases (42,572 locally-acquired, 209 deaths) [75,77]. Most of these cases (53.4%) occurred in Tainan city and 44.5% in Kaohsiung city located in southern Taiwan [75,77].

Outbreaks of dengue usually peak in the summer and autumn [101]. Despite repeated dengue outbreaks caused by importation to the southern part of the island, Taiwan is not currently considered dengue-endemic [72]. Based on a study of 9,939 cases of dengue fever and DHF from 2000 to 2007, the duration of illness for dengue fever and DHF (excluding fatalities) was 8.4 and 10.8 days, respectively [102]. Active surveillance from 2004–2007 reported a DENV seroprevalence of 1.1% (of 42,150 total cases) and 77% of cases as primary dengue [72].

### Economic Burden of Dengue

Estimates of the economic burden of dengue are calculated by adding the direct and indirect costs. The direct costs consist of health care costs such as those related to the provision of health care, non-health care costs such as those related to consumption of resources like transport, and household expenditures. The indirect costs are those associated with the loss in productivity from illness and death [103]. A robust assessment of the economic burden helps to i) identify information gaps, research needs, and refinements to the national statistical reporting systems; ii) argue that policies on dengue control and prevention should be given a high priority on the public health policy agenda; and iii) provide a baseline measure to determine the cost effectiveness of dengue policies and programs [104]. However, few studies have assessed the economic burden of dengue [105]. The estimation of the true extent of dengue, its incidence, and its associated costs have substantial uncertainty, partly because of unreported and unrecognised apparent DENV infections [8].

In 2013, there were an estimated total of 58.40 million symptomatic DENV infections globally, including 13,586 fatal cases [8]. The annual global cost of dengue illness averaged US$8,900 million. Globally, 18% of cases are admitted to the hospital, 48% remain ambulatory, and 34% do not seek medical care. Costs per case are generally higher in HICs, especially in Australia and the US [8].

#### Queensland, Australia

The average work time lost was 10.5 days among people who recalled a dengue-like illness during the 1993 dengue epidemic in Charters Towers, Australia [106]. From 1990 to 2008, the cost of lost work and the control cost averaged an estimated US$36.18 million (2.01 million per annum) [107]. However, this calculation does not include physical and psychological suffering. A delayed response to dengue outbreaks of 4–6 weeks would result in 86 times (or US$13 million in 2003) and 346 times (or US$382 million in 2009) higher dengue illness costs versus a scenario with active surveillance and response within 2 weeks, respectively [108]. Therefore, effective dengue surveillance and response lowers costs associated with dengue.

#### Florida, US

Cost has not been estimated in Florida specifically, but estimates have been made for the US and many central and south American countries [109]. Shepard et al. (2011) estimated the aggregate annual total cost of dengue in the Americas to be about US$2.1 billion for the period 2000–2007 [109]. The US had the highest estimated average total cost per dengue case (ambulatory cost added to hospitalized cost = US$19,413) among the selected countries.

#### Singapore

Among the 12 countries analysed by Shepard et al. [4] (Bhutan, Brunei, Cambodia, East Timor, Indonesia, Laos, Malaysia, Myanmar, the Philippines, Singapore, Thailand, and Vietnam), which did not include the US, Australia, or Taiwan, the predicted values of direct and indirect unit costs per dengue case were the highest in Singapore (direct costs: $2,455, indirect costs: $1,821 in 2010 US dollars). In Singapore, Carrasco et al. [6] investigated the cost-effectiveness of future vaccination programs and estimated that, from 2000 to 2009, the average economic impact of dengue illness in constant 2010 US dollars ranged from $0.85 billion to $1.15 billion, of which control costs constituted 42%–59% [6].

#### Taiwan

In Taiwan, it cost about US$227 (2011) to treat a patient with dengue fever [110]. Tseng et al. (2009) estimated the economic impacts of climate change on dengue in Taiwan. The probability of being infected by DENV due to climate change ranges from 12% to 43% to 87%, which represents low, mid, and high probabilities when the temperature increased by 1.8°C [111]. People would pay US$22.8, US$102.3, and US$162.28 (in 2008), respectively, per year in order to avoid the increased probabilities of being infected with DENV. Visitor numbers for September 2015 were 50% lower than the same period in 2014, due at least in part to dengue [112].

## Drivers of Dengue Transmission

Nonclimatic factors include the epidemiology, ecology, and distribution of the vector, socioeconomic, and environmental factors; climatic factors include weather variability, climate change, and extreme events. These factors are discussed in detail in the following sections.

### Vector Distribution

*Ae*. *aegypti* is most prevalent in highly urbanized areas, whereas *Ae*. *albopictus* is present mainly in rural, suburban, and vegetated urban areas [113–115]. Abiotic factors including urban landscape, temperature, humidity, and rainfall are strongly associated with the abundance of both species [116]. Displacement of *Ae*. *aegypti* by *Ae*. *albopictus* because of resource competition among larvae and crossmating between *Ae*. *albopictus* males and *Ae*. *aegypti* females might reduce the overall risk of dengue transmission [115,117,118]. Continued vector surveillance is crucial in susceptible areas currently free of *Ae*. *aegypti* and *Ae*. *albopictus*.

*Ae*. *albopictus* and *Ae*. *aegypti* are present in Queensland, Florida, Singapore, and Taiwan. The former is not yet established in Monroe County, Florida [119] or in mainland Queensland, where *Ae*. *aegypti* is prevalent. In Singapore, *Ae*. *aegypti* predominates [120]. In Taiwan, *Ae*. *aegypti* only occurs south of the Tropic of Cancer (23°26′N 120°8′E, Chiayi and Hualien Counties), and *Ae*. *albopictus* is distributed throughout the country and below an elevation of 1,500 m above sea level [98].

The movement of *Ae*. *albopictus* from the Torres Strait Islands, Queensland, to the mainland, with subsequent potential expansion southwards, could increase the area that is receptive to DENV epidemics [121,122] and also expand the area receptive to transmission of other viruses including chikungunya and Zika. This is also the case in central, northern, and eastern parts of Taiwan, where only *Ae*. *albopictus* exists and outbreaks occur rarely. *Ae*. *aegypti* is thus the main vector, but *Ae*. *albopictus* can transmit dengue in the absence of *Ae*. *aegypti* [71]. The number of locally acquired cases is higher in the southern than in other parts of Taiwan, despite a lower number of imported cases. Therefore, the presence of *Ae*. *aegypti* may be essential to outbreak initiation. In Southern Florida, [116,119] *Ae*. *aegypti* is more abundant in the early dry season than in the late wet season and can spread during the early wet season in the absence of *Ae*. *albopictus* [116].

### Epidemiology and Ecology of Transmission

High DENV virus replication rates are associated with a high vertical transmission rate in mosquitoes [123]. Fewer *Ae*. *aegypti* than *Ae*. *albopictus* progeny are infected vertically, although the latter species may sustain DENV during interepidemic periods [123,124]. Vertical transmission of DENV-1 from infected *Ae*. *aegypti* female mosquitoes to their eggs may have served as an interepidemic reservoir between outbreaks in successive years in Key West, Florida [125], as could low-level horizontal transmission, especially if not detected. In Taiwan, although continuation of outbreaks through winter is rare, three overwinter outbreaks occurred from1987–2010: 100,000 cases from 1987–1988; more than 5,000 cases from 2001–2002; and more than 1,400 cases from 2009–2010 [101].

Many dengue importations into Australia are presumably undetected or unreported [120]. In Charters Towers, Queensland, subterranean breeding (e.g., in septic tanks, sump pits, telecommunication pits, mine shafts, and wells) contributed significantly to *Ae*. *aegypti* populations and therefore DENV-2 transmission during the 1993 outbreak [126]. Many larval sites are cryptic and difficult to locate, particularly subterranean sites and elevated sites such as rainwater tanks and roof gutters [127]. Subterranean sump pits and wells can support large populations of *Ae*. *aegypti*, especially during the winter season when surface containers are dry [127–129]. A reduction in the number of large water storage containers likely reduced *Ae*. *aegypti* distribution in the past [130].

In Singapore, refuse is commonly an *Aedes* oviposition site [131]. The main breeding habitats in homes are domestic containers, flower pot plates, ornamental containers, and plants, and in public areas discarded receptacles, closed perimeter drains, gully traps, Housing and Development Board (HDB) corridor drain/gullies, and plants [132].

### Increased Movement and Proximity to Endemic Regions

With increased transport of materials and people, the potential for vector transportation and virus transmission and spread has increased substantially [133]. In Queensland, Florida, and Taiwan, the arrival of viremic travelers is currently necessary for local transmission. In Australia, dengue is now more often diagnosed than malaria in ill travelers who have returned from tropical regions (except Africa) [134]. International arrivals to Townsville and Cairns airports commenced in 1980 and 1984, respectively, and consequently the number of travelers from dengue-endemic countries has increased significantly [135]. Because Queensland, Florida, Singapore, and Taiwan are popular tourist destinations, this increases the likelihood of virus introduction and subsequent local transmission.

During the 1997–1999 epidemic in North Queensland, the movement of viremic individuals facilitated multiple initiation foci [23]. Today in the US, Miami, Florida is the most used airport in terms of the number of international passengers boarding American carriers [136]. Key West is a tourist destination with more than 2 million visitors annually [137]. In 2013, Changi Airport, the main airport in Singapore, handled more than 53.7 million passengers with an annual growth rate of 4.7% [138,139]. About 203 million travelers and 68 million conveyances were reported to cross the border between the southern Malaysian state of Johor and Singapore in 2013 [140]. During the second largest DENV outbreak in Taiwan in 2014, a total of 44.8 million passengers (an increase of 12.6% over 2013) were traveling on international, cross-strait, and transit flights [141]. As a travel hub, Singapore [56] and Taiwan experience continuous importation of DENV.

Proximity to endemic regions and the increased number of dengue cases in neighboring countries presents a significant risk for countries with endemic vectors [142]. In Australia, among cases with a known source, 23% originated overseas from 1991 to 1999 and 64% from 2000 to 2012. Between 1995 and 2011, the main sources of dengue importation in Australia were from neighboring dengue-endemic countries such as Indonesia (24.6%), Papua New Guinea (23.2%), Thailand (13.4%), East Timor (8.9%), and the Philippines (6.7%) [33]. Florida is also very close to dengue-endemic countries in the Caribbean (e.g., Puerto Rico), Central America (Honduras), and South America (Brazil) and is the top US destination for Puerto Rican migrants [143]. In Singapore from 2000 to 2014, the neighboring dengue-endemic countries Indonesia and Malaysia were the two main sources of dengue importations, contributing 38% and 26%, respectively, of the total imported cases in Singapore [50–53,55,58,60–65,68]. Virus importation by tourists and migrant workers (e.g., from China in 2002 [144]) from dengue-endemic countries and decreasing herd immunity have contributed significantly to the failure of dengue control in Singapore [145]. Over the past two decades in Taiwan, imported cases have played an important role in the major outbreaks of dengue [146]. Between 1981 and 2010, imported dengue cases primarily involved travellers from the Philippines, Vietnam, Thailand, and Indonesia [101].

### Housing Structure, Urbanization and Population Growth

In many tropical regions, urbanization, lack of dependable water systems, overcrowding, and poor housing contribute to large dengue epidemics [80]. This is less of a problem in HICs. However, in North Queensland and Key West, Florida, dengue transmission has been associated with old unscreened housing, allowing access to *Ae*. *aegypti* [147,148]. Typical “Queenslander” houses, designed with architectural characteristics suitable to a tropical or subtropical climate such as large sprawling timber structure on stumps, and an extensive deep shaded verandah, are being replaced by new apartment blocks designed with screening and air conditioning in Cairns and other Far North Queensland centers. These should limit dengue transmission [127] because avoidance of mosquito bites effectively prevents transmission [149]. In Queensland, sites such as backpacker hostels are identified as “ignition” or starting points, whereas centers where communities congregate, such as schools and churches, are identified as “dissemination” points [23] from which the virus may spread to multiple geographic foci [23].

Between 2006 and 2011, Australia, particularly Queensland, experienced rapid population growth [150,151], with a total of 121,000 new Queensland residents over the five years [152]. The Queensland average annual population growth was 2%, [150,151] primarily driven by overseas immigration [153]. The 2006 Census shows that the main countries of origins of new Cairns residents were the United Kingdom (UK), Japan, Philippines, Korea, and Papua New Guinea, whereas other regional areas, such as Townsville and Mackay, feature migrants prominently from the UK, India, the Philippines, and South Africa [152].

**Fig 3:**
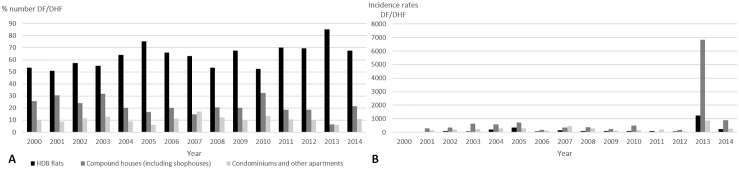
Percentage and incidence rate of reported indigenous dengue fever and DHF cases by housing type for Singapore residents from 2000–2014.

Singapore is highly urbanized with a population of 5.47 million in 2014, an increase of 1.3% from 2013, and a population density of 7,615 persons per km^2^ [154]; high population density increases dengue risk. In Singapore, about 80% of the houses are managed by the HDB [155]. Before the mid-2000s, most houses had bamboo pole holders outside the kitchen windows that were used to dry laundry, but also provided good breeding grounds for mosquitoes [156]. Now, the bamboo poles can be covered [157], supported from both ends, or replaced by a laundry rack. Although these changes should help prevent the breeding of *Aedes* mosquitoes, no studies have confirmed this. Over the period 2002–2014, residents in HDB compound houses, free standing properties (including shops and houses), and condominiums constituted 70%, 19%, and 11%, respectively, of the dengue cases. Incidence rate was higher in those living in free standing properties [51,53,55,58,60–65,68] (Fig 3).

In Taiwan, from 1998 to 2002, a higher level of urbanization was also associated with increased risk of dengue fever at the township level [158]. Poor environmental hygiene usually goes with rapid urbanization, which tends to increase vector breeding. It is unclear why the 2015 outbreak clustered intensively in Tainan city, but a persistent problem of illegal dumping of garbage in the local parks possibly contributed through pooling of water and consequent mosquito oviposition [159].

### Weather Variability, Climate Change, and Extreme Events

Climate change may contribute to the emergence and re-emergence of dengue through its interactions with biotic and abiotic factors. However, due to the complex interaction of climate and other factors, the magnitude and direction of changes in incidence are difficult to predict.

Australian annual average daily mean temperatures have increased progressively by 0.9°C since 1910, and the frequency of extreme weather has changed, with more heat and fewer cool extremes [160]. Substantial warming has occurred in the three oceans (Pacific, Indian, Southern) surrounding Australia [161]. By 2070, Australian temperatures are projected to rise by 1–5°C, depending on global CO_2_ emissions. Consequently, climate sensitive diseases are expected to increase in incidence [162].

In southern Florida, climatic factors may be the cause of competition between *Ae*. *albopictus* and *Ae*. *aegypti* [163]. The presence of *Ae*. *albopictus* was significantly lower after the dry season than during the wet season, whereas the *Ae*. *aegypti* presence was consistent [163]. However, local coexistence of these species is possible when a warm dry climate favors *Ae*. *aegypti* and reduces the competition from *Ae*. *albopictus* by increasing mortality of *Ae*. *albopictus* eggs [163]. In the laboratory, dry periods cause disproportionately greater mortality of *Ae*. *albopictus* eggs compared to *Ae*. *aegypti* eggs [163].

In Singapore, from 1972 to 2014, the annual mean temperature has increased from 26.6°C to 27.7°C, and rainfall has become more intense [164]. Temperature, relative humidity, and El Niño Southern Oscillation were significantly and independently associated with dengue cases in Singapore [165]. Moreover, in Singapore and Taiwan, rainfall, temperature, and dengue fever incidence are positively related [166,167]. Health officials in Singapore reported a surge in dengue cases in December 2015, which coincided with warmer weather associated with the El Niño phenomenon [69], although no study has confirmed this.

In Taiwan, dengue transmission typically begins with importation in the summer and ends in the winter. The combination of its subtropical climate, neighboring hyperendemic countries, the hot rainy summer (average temperature, 28°C) and dry cold winter (average 20°C), and the coexistence of two vectors facilitates dengue transmission in the summer and autumn [72]. In 2007, after an initial wave of dengue infections in Taiwan, the temperature was reduced by two typhoons, and fewer cases were reported. However, in early autumn, the subsequent increase in moisture and temperature caused a second wave of dengue fever [168]. The exact causes of the large outbreak in Kaohsiung, Taiwan, in 2014 are not known, but it is believed that higher mean temperatures than in previous years (+0.5°C–1.3°C from June–September 2014) contributed [75]. The causes of the intensive and clustered 2015 outbreak in Tainan are also poorly understood. Large areas of standing water accumulated in some parks after flooding and only drained for two to three weeks may have been the primary breeding areas for mosquitoes that sustained the epidemic [159].

Drought together with population and economic growth have caused anthropogenic environmental changes. These include limited water supply relative to demand in Queensland cities, despite flooding, which has led to an increase in rainwater tank usage. Regulations and guidelines mandate or recommend measures to prevent mosquito breeding in tanks, including flap valves and screens with mesh size of no more than 1 mm [169]. Nonetheless, an increase in domestic water storage could increase future mosquito populations and consequently dengue risk [170]. In North Queensland, dengue transmission is also associated with unscreened rainwater tanks. Drought and consequent changes in water storage practices are projected to increase the *Ae*. *aegypti* range [170]. Under climate projections, shade-dependent evaporation interacts with container size and biological characteristics, such as resistance to cold and egg dessication, to determine geographic range for *Ae*. *aegypti* [171]. Simulation modelling indicates that climate warming may lead to increases or decreases in dengue vector abundance, depending on the level of carbon emissions [172].

## Responses and Challenges

### Public Health Responses

#### Queensland, Australia

Since the 1992–1993 dengue epidemic, public health authorities in north Queensland have intensified surveillance, control, and education programs (e.g., the “Tip it, Store it, Throw it” message), and the development and use of predictive tools [27]. Strategies are applied at three different activity levels: ongoing prevention; response to sporadic cases; and outbreak response [127]. When a case is notified, officials interview the patient and complete a case report form [27]. Control activities include larval and adult control within a 200 m radius of the case residence and high-risk contact areas [27]. The use of indoor residual spraying targeting resting sites of *Ae*. *aegypti* has been shown to significantly decrease dengue transmission in Cairns [173] and has become the primary adulticiding method used. Regulation of water storage to minimize mosquito breeding is particularly important for detecting and eliminating the most productive sites [174].

#### Florida, US

Following the introduction of West Nile virus in the northeastern US in the summer and autumn of 1999, the US Centers for Disease Control and Prevention (CDC) and the Department of Agriculture cosponsored a meeting of experts to develop guidelines for the surveillance, prevention, and control of West Nile virus and other arboviruses [175]. Dengue became a nationally notifiable disease in the US in 2009 because of concerns about the increasing number of importations and the consequent risk for local transmission and transfusion transmission [176]. During the Key West outbreaks, the Florida Keys Mosquito Control District (FKMCD) used all means available to reduce the populations of *Ae*. *aegypti*. Response strategies in Key West included public meetings and communication, education at schools, and tourism council press releases [42]. Tourism, public health, and vector control officials collaborated in response. Health department personnel conducted outreach visits to clinicians. Vector control activities include spraying adulticides within a 200 m radius around homes of case-patients, outdoor residual and spatial insecticide treatments, door-to-door campaigns to educate the public, and finding and eliminating mosquito breeding sites [42]. Florida law mandates inspections and the elimination of breeding sites; however, fines are not usually imposed and violations are frequent [42]. Since the implementation of response strategies, autochthonous dengue infections still occur in Key West, and a 2012 survey of the decision makers involved in the control of the outbreak revealed the need to focus prevention strategies on educational campaigns [177].

#### Singapore

The vector-control programs instigated in the late 1960s have progressively reduced dengue transmission and thus herd immunity. As a consequence, the incidence of dengue has surged since the 1990s, mainly in young adults [178]. Low herd immunity and a shift from domestic to nondomestic transmission (e.g., schools, hospitals, and workplaces) are the main issues in Singapore [179]. The number of potential breeding sites in nondomestic settings is large, and these are poorly identified and localized. The National Environment Agency (NEA) adopts a multipronged approach to control dengue comprising preventive surveillance and control, public education, and community involvement, enforcement, and research. The strategies include active surveillance in areas prone to dengue or where mosquito populations are high, and source reduction when cases are reported [180].

In Singapore, pyrethroids were commonly used for fogging in the past and are still commonly used as insecticide by the pest control industry. Consequently, *Ae*. *aegypti* has become pyrethroid-resistant in Singapore [181]. Crossresistance between dichlorodiphenyltrichloroethane and pyrethroids occurs [182]. Therefore, viable alternatives including biological control are urgently needed. However, biocontrol approaches might impose evolutionary selective pressures on the virus and vectors. The viral diversity resulting from the increase in dengue transmission over recent years and reduced mosquito population because of vector control may also have contributed to selection pressure and to the evolution of a virus lineage with better fitness; i.e., DENV that would spread better through *Ae*. *aegypti* population [91]. Although new environmentally friendly biocontrol strategies show promise, an improved understanding of the biology and ecology of the vectors is crucial [130].

The Singapore government introduced a fine in 2005, which doubled in 2008, for all homes found to be breeding mosquitoes [183]. For example, in 2012, 900 mosquito-breeding offenses were detected on construction sites, with 626 being first-time offenders [184]. To implement an effective vector-control program, more officers were recruited to conduct routine checks of homes within dengue-active zones in 2013. When residents did not respond to notices, the NEA used its legislative powers to gain entry to homes [185]. The NEA fined a town council US$160 (in 2013 US dollars) for letting mosquitoes breed in water tanks. Town councils which break the law twice could be fined up to US$1,500. As of August 2014, the NEA has inspected 1.9 million houses or premises and has deployed more than 1,000 gravitraps in dengue clusters for mosquito-control purposes, in addition to regular fogging and space spraying in certain hotspots. The NEA has prosecuted 14 contractors and issued 62 stop-work orders [186]. Since March 14, 2016, the owners of homes found to be breeding mosquitoes are fined US$148 (in 2016 US dollars), regardless of whether the home is within a dengue cluster [187].

Since 2015, the NEA has also increased its epidemiological investigation to identify potential sources of infection other than registered residential addresses, such as workplaces and areas of congregation. NEA improved its dengue control by i) increasing inspections by creating dedicated construction site teams, ii) publishing the list of sites issued with stop-work orders on the dengue microsite to serve as a deterrent to contractors, iii) requesting contractors to put in place a temperature-screening regime to identify cases earlier, iv) encouraging application of insect repellent and, v) for dengue-infected workers, to sleep under bed nets or in air-conditioned sick bays to prevent further transmission [188].

#### Taiwan

Before 2009, the Taiwan Centers for Disease Control (TCDC) used only a clinical case definition for reporting dengue, which may have led to over-reporting. The TCDC’s current dengue case definition, established in 2009 and in use since, requires laboratory confirmation for reported cases [189]. Dengue must be reported to TCDC within 24 hours [72]. A central command center was established to provide resources and personnel from various ministries (Education, Interior, National Defense) and agencies (department of health, environmental protection administration, government information office). The aim was to foster close cooperation with local government units and public education to reduce vector breeding. These efforts have been associated with reduced case numbers from 2010 to 2012 [70]. However, it is difficult to confirm this trend because of the short period.

When *Aedes* larvae are found or when an individual refuses control measures, a significant fine is imposed [190]. Taiwan had also established airport fever screening [191]. Questionnaire screening was used from 1998 to 2002, but following the 2003 severe acute respiratory syndrome outbreak, fever screening at the airport was added. Fever screening at the airport has been successful in identifying 45% of 542 known imported dengue cases with fever [191]. However, the intensity of local transmission is not proportional to the number of imported cases [191]. Pyrethroids are also often used to control *Ae*. *aegypti* and thereby suppress epidemics in Taiwan. However, *Ae*. *aegypti* has also become pyrethroid-resistant in Taiwan [192].

### Community Involvement

Singapore has in place community-based programs for the removal of potential breeding habitats [193], which involve the community in mosquito control through environmental management, health education, and community ownership (“bottom-up” approach) (e.g., “Do the Mozzie Wipeout” Campaign, “Do the 5-Step Mozzie Wipeout”) [194]. This is essential for avoiding community complacency. However, the community-based approach can be slow or demotivating and, as a consequence, unsustainable [195]. Bottom-up and top-down approaches should be combined [195].

In addition to the proactive measures against mosquito-breeding sites, efforts have been made to increase opportunities for people to interact through social media focused on dengue transmission. Examples include “Stop Dengue Now” and “Amy Khor” in Singapore and “Follow the Moz” or “Eliminate Dengue” Facebook pages in Queensland. Unfortunately, the latter two are no longer updated. Although the use of these forms of online social media cannot provide a solution for reducing dengue transmission, it empowers citizens by giving them easy access to knowledge and by proposing a form of participatory surveillance involving mutuality and sharing.

Singapore citizens actively voice opinions on social media about dengue. For example: “(…) Each time an NEA officer knocks on my door, I cooperate with them but at the same time, I wonder whether the door-to-door checks aren't a waste of time when my HDB neighbourhood is strewn with litter such as empty cigarette packs, plastic bags, and dirty food containers. Each piece of litter is a breeding ground (…)” [196], “Residents must take greater responsibility to rid their homes of mosquitoes breeding. I have often witnessed households not responding to NEA officers knock (…)” [197]. Local residents in Singapore also have the opportunity to volunteer in efforts to control dengue. Dressed in yellow vests and red shirts, they go from door to door encouraging their neighbors to assist in controlling dengue [198].

A smartphone application designed to encourage citizens to identify and report potential breeding sites of *Aedes* mosquitoes is also available (e.g., TrashWatch, http://www.vectoranalytica.com/inc/products/trashwatch/). However, some countries, including Australia, do not have access to this application.

### Challenges

From 2012–2013, the Queensland Government budget cuts impacted hospital and health services in Queensland, mainly in Metro North (–22.5 million Australian Dollars) and Metro South (–18.8 million) areas in Brisbane, Gold Coast (–9.2 million), Townsville (–7.8 million), Sunshine Coast (–7 million), and Cairns (–6.5 million) [199]. These cuts have impaired dengue prevention and control programs [200]. Moreover, unlike the US, Singapore, and Taiwan, Australia lacks a single center, such as the CDC, for dissemination of information to the public, relevant organizations, and government agencies. In the US, public health budget cuts have also impaired vector surveillance and control programs [201]. In 2011, the Florida Department of Health Infectious Disease Control branch budget was reduced by US$3.8 million with consequent loss of 172 full-time positions in health departments [202]. In early 2016, the President of the US asked Congress to allocate US$1,900 million to surveillance and control of the Zika virus, *Ae*. *Aegypti*, and *Ae*. *albopictus*, but, to date, lawmakers had not acted [203]. In Florida, the mosquito control budget varies greatly between counties. Lee County, home to Fort Myers and 700,000 people [204], funded by local property taxes, spends $16 million a year and has a staff of 88, ten helicopters, and four propeller planes for spraying [205]. In contrast, Vasquez, an entomologist and director of the mosquito-control district of Florida’s Miami Dade County, Florida’s biggest county with 2.6 million people, spends a $1.6 million budget a year, enough for 15 employees. Moreover, his inspectors are not always welcomed by the community and have to deal with denial, angry homeowners, and, sometimes, local politics [203].

Early recognition and notification of dengue cases [33] and timely initiation of appropriate supportive care is critical to reduce medical complications and mortality among patients with severe forms of dengue [206]. In Florida, at least until 2010, medical and public health practitioners tended to have limited experience with dengue, and the failure of diagnosis and reporting led to delayed detection of DENV introduction [7]. In the Key West 2009–2010 outbreak, initial cases were attributed by local physicians to nonspecific viral illness without consideration of dengue [207]. Although Singapore generally has an efficient clinical service, hospitals are sometimes crowded [208].

Because mosquitoes sufficient to infect an entire neighborhood can breed in a small number of negligently maintained properties, citizen cooperation is indispensable [209]. However, cooperation between citizens and public officials is often lacking, and there may be outright obstruction of mosquito-control efforts. Unfortunately, control is often seen as the exclusive responsibility of local and state mosquito-control agencies [89].

Transfusion-transmitted dengue is rare [210], but no licensed donor-screening test for DENV is available worldwide. Consequently, many blood donations are unable to be collected during epidemics [211]. Rigorous selection of donors offers protection but is labour intensive, costly, may affect sufficiency of supply, and may not be possible.

## Lessons and Recommendations

### What Do These Areas Have in Common?

The countries and states presented in this review have in common i) a competent mosquito vector, ii) introduction of DENV by travelers, iii) increased local transmission of dengue coinciding with population growth and increased mobility, iv) recent budget cuts impacting public health services, and v) a largely nonimmune population.

The four countries and states have areas where *Ae*. *aegypti* is established. Outbreaks rarely occur where only *Ae*. *albopictus* is present (e.g., central, northern, and eastern parts of Taiwan and the Torres Straits Islands in Australia). The four countries and states are surrounded by dengue-endemic countries and have air connections with dengue-endemic countries. In Queensland, Florida, and Taiwan, the arrival of viremic travelers is a prerequisite for local transmission. Both Taiwan and Singapore are highly populated and urbanized, increasing dengue transmission risk. Both Florida and Queensland have experienced past budget cuts that affected public health services. Florida and Queensland are particularly at risk for autochthonous dengue outbreaks due to its largely nonimmune populations.

### What Is Unique?

The countries and states presented in this review also display unique features, such as i) their dengue epidemic/hyperendemic status, and consequent mortality rate, ii) the frequency, amplitude, and duration of outbreaks, iii) their vector control program and community engagement.

Dengue is hyperendemic in Singapore and Taiwan, but epidemic in Queensland and Florida, and the rarity or absence of serious outcomes in Australia and Florida may be due to the infrequency of consecutive infections with different serotypes. DHF and deaths are rare in Australia, and absent in Florida, but occur often in Singapore and Taiwan. Mortality is usually linked to delayed provision of supportive treatment or presence of comorbidities [212].

The recent epidemiology of dengue in Singapore was characterized by a 5–6-year cycle [9,56]. However, since 2013 in Singapore, the incidence rates have markedly increased, exceeding 10,000 cases each year. Dengue transmission in Florida is still reasonably limited, and the majority of dengue cases reported are travel-associated cases [88].

In Queensland, the “Tip it, Store it, Throw it” message is now being heard by the local community according to Queensland Health’s Cairns acting director of tropical public health services [35]. In Far North Queensland, the number of dengue fever cases in 2015 was 33% lower than the total dengue cases in 2014 [36]. Compare to the other three countries and states, Singapore citizens have more opportunities to engage on social media about dengue, to be aware of the problem, and thus to be proactive. However, despite the hard work of contractors engaged by the NEA to clean out certain private estates and public areas throughout Singapore, littering remains an environmental and social problem linked to economic and health issues [131].

## Management Options in the Near Future

This review summarises key determinants for dengue transmission, which could be better managed to help reduce transmission (Table 2). Some are relevant for many countries and states (e.g., proximity to endemic countries, population growth and movement, lack of awareness, and engagement of residents/tourists) and some are country/region specific (e.g., high level of urbanization, budget cuts, housing structure).

**Table 2:** Key determinants for ongoing dengue burden in Queensland (Australia), Singapore, Taiwan, and Florida (US) and recommendations.

	Key determinants	Key recommendations
**Queensland, Australia**	proximity to endemic countries dramatic increase in imported cases via arrival of viremic residents and tourists in the last ten years absence of a national disease control network low herd immunity transmission associated with old unscreened housing budget cuts cryptic sites (subterranean, elevated) can produce large numbers of *Ae*. *aegypti* population growth and movement increase in rainwater tank usage lack of awareness and engagement of residents/tourists	In Queensland and Florida, the Key West/Martin counties outbreaks show that localized transmission, where *Ae*. *aegypti* is abundant, can occur. These suggest that vector surveillance and control programs need to be sustained for rapid identification and control of outbreaks. A communicable disease center in Australia is critical for managing and lowering future disease risks. The center would play a key role in engaging state and territory health departments, aiding rapid response to potential threats. The US and European CDCs would be appropriate examples for such a national structure. To be successful, dengue control programs for HICs must also consider the epidemiology of dengue in other endemic countries that may increase virus importations. “Top-down” and “bottom-up” approaches should be combined. Community engagement in reducing vector breeding is crucial, and community members should be encouraged to cooperate with vector control agencies. Control the cryptic larval habitat (elevated and subterranean sites). Control that targets the adult mosquito vector is important. Travel-related risks need to be better managed and incorporated in national strategies for nonendemic countries that experience, or are at risk for, epidemics. Tourism bodies need to be involved in disease prevention in order to diminish possible opposing viewpoints. Education of the public and the medical profession is central to prevention. To avoid institutional memory lost when key employees leave, (“Brain-Drain” effect), transition to their replacements should be prepared to preserve this information. A robust assessment of the economic burden (direct and indirect costs) of dengue infections is highly needed for those countries to justify investing in dengue control programs.
**Florida, US**	proximity to endemic countries dramatic increase in imported cases in the last ten years transmission associated with old unscreened housing (in Key West) low herd immunity budget cuts usually fines not imposed when breeding sites found population growth and movement lack of awareness and engagement of residents/tourists	
**Singapore**	proximity to other endemic countries all serotypes circulating and high diversity low herd immunity resistance to pyrethroid insecticides in dengue vectors shift from domestic to nondomestic transmission virus importation by tourists and migrant workers important air traffic/travel population growth and movement high level of urbanization unbalanced health care system lack of awareness and engagement of residents/tourists/migrant workers (e.g., excess of litter)	
**Taiwan**	proximity to endemic countries important air traffic/travel population growth and movement high level of urbanization lack of awareness and engagement of residents/tourists (e.g., excess of litter)	

### Vaccine

The world’s first dengue vaccine, Dengvaxia (CYD-TDV) by Sanofi Pasteur, first registered in Mexico early December 2015, and later approved by the Philippines and Brazilian authorities, has provided some hope [213]. CYD-TDV is a live recombinant tetravalent dengue vaccine that has been evaluated as a three-dose series on a 0/6/12 month schedule in Phase III clinical studies, and is for use in individuals aged 9–45 years living in endemic areas [213]. This vaccine is currently being reviewed by around 20 countries in Asia and Latin America [214]. The Phase III trials showed a variation in vaccine efficacy according to the age at vaccination and serostatus before vaccination [213,215]. Five additional vaccine candidates are under evaluation in clinical trials [213]. Recommendations concerning the CYD-TDV vaccine will be published by the World Health Organization in July 2016 [213].

### Vector and Virus Control

In 2012, the FKMCD announced that it had partnered with the British firm Oxitec to release large numbers of genetically modified *Ae*. *aegypti* males in Key West, Florida [89]. This project was considered but rejected by some residents, and the use of genetically modified male mosquitoes in Key West is still in negotiations and is awaiting the US Food and Drug Administration approval [89,216]. The re-emergence of dengue in Florida as well as the threat posed to the US from other emerging mosquito-borne arboviruses (e.g., chikungunya, Zika viruses) emphasizes the need for strong vector-borne disease surveillance and mosquito-control infrastructure to rapidly identify and control outbreaks of dengue or other mosquito-borne diseases.

Transinfection of *Ae*. *aegypti* with the endosymbiotic bacterium *Wolbachia pipientis* is undergoing field trials in Australia, Indonesia, Vietnam, Brazil, and Columbia [217]. Some strains of *Wolbachia* can reduce *Ae*. *aegypti* lifespan and reproduction and thus interfere with DENV replication and transmission [218,219]. The virulent *Wolbachia* strain *w*MelPop has been shown to cause widespread degeneration of tissues (brain, retina, muscle) and early death in *Ae*. *aegypti* adults [220] and eggs [221] but failed to establish following field releases. Releases of the more benign *w*Mel strain led to establishment in seven Cairns suburbs (infection frequency > 90%), and this strain has persisted for at least five years [222,223]. A field trial was later launched in Townsville, North Queensland, in October 2014 [224]. In Singapore, the NEA’s Environmental Health Institute has tested the use of *Wolbachia* bacteria in the laboratory, but there have not been field trials. Reliance on insecticides for dengue control has been excessive [225], and these compounds are costly and sometimes ineffective in urban areas.

### Modeling and Geographic Information Systems

Such deficiencies and limitations of current vector-control strategies have necessitated the development of predictive mathematical, statistical, or spatial models for dengue early warning systems [225–228]. The development of a climate-driven spatiotemporal prediction model and the use of geographic information system in dengue surveillance are essential to inform disease prevention and control interventions. Yu et al. (2011) [166] proposed a spatiotemporal climate-based model of early dengue fever warning in southern Taiwan, based on stochastic Bayesian Maximum Entropy analysis. The analysis provides the required “one-week-ahead” outbreak warnings based on spatiotemporal predictions of dengue fever distributions, that can be used by the Taiwan Disease Control Agency to timely identify, control, and prevent dengue fever transmission [166]. Hii et al. (2012) [229] developed a weather-based dengue forecasting model that allows warning 16 weeks in advance of dengue epidemics in Singapore with high sensitivity and specificity. The authors demonstrate that models using temperature and rainfall could be simple, precise, and low cost tools for dengue forecasting and could be used to enhance decision making on the timing and scale of vector control programs [229]. However, these tools also have limitations because the factors used for prediction may interact nonlinearly [230]. Models may also under- or overestimate the projected incidence, and they are often regionally specific and consequently not suitable for global projections. Similarly, global models [231] are not necessarily appropriate for small-scale projections. Geographic information system technology is a useful tool for creating a user-friendly map to show the physical location of dengue cases from authoritative sources such as the NEA (e.g., Outbreak, http://outbreak.sgcharts.com/). Finally, traveller behaviour, vector evolution, biocontrol impacts, and other important risks are unpredictable.

## Conclusion

Changes in climatic conditions, patterns of human settlement, movement, and population density, distribution of the two main mosquito vectors, and water-management technologies have all influenced the occurrence of dengue outbreaks since the mid-20th century. The competitive relationship between the two main vectors, *Ae*. *aegypti* and *Ae*. *Albopictus*, may lead to future changes in the epidemiology of the disease. This review has considered dengue transmission in Queensland in Australia, Singapore, Taiwan, and Florida in the US. We have identified important epidemiological issues in these regions, such as population growth in the four settings, increases in local transmission despite control efforts in Singapore and Taiwan, increased human mobility from neighboring endemic countries (especially in Taiwan and Singapore), lack of citizen engagement in the four settings, and highly populated urban areas (in Taiwan and Singapore). Budget cuts in health and the current absence of a practical vaccine contribute to an increased risk.

In the HICs discussed, dengue has become an increasing challenge, despite active vector control programs. However, it would be naïve to assume a single cause, or even a small set of causes, is adequate to explain the change in dengue epidemiology in any or all of the countries discussed. Host, agent, and environmental factors all interact. These include demography, travel, strain variation, and changes in housing and the global environment. Because the factors affecting dengue incidence and transmission are diverse, a single model for disease control is unlikely to be applicable in all settings. Assuming ongoing global economic growth, other countries where *Ae*. *aegypti* and/or *Ae*. *albopictus* are established (e.g., France and Japan) may, in future experience, regulate dengue transmission [232,233]. The epidemiology of dengue will continue to change. Although *Ae*. *aegypti* is the dominant urban vector, *Ae*. *albopictus* can also cause outbreaks, especially in less tropical areas. Flexibility, ingenuity, and imagination will be required to control dengue in the face of these challenges.

**Key Learning Points**

- Increased human mobility from neighboring endemic countries.
- Lack of awareness and engagement of residents/tourists/migrant workers (excess of litter is a recurrent issue).
- Resistance to pyrethroids insecticides.
- Budget cuts in health.

**Top Five Papers**

1. Bhatt S, Gething PW, Brady OJ, Messina JP, Farlow AW, Moyes CL, et al. The global distribution and burden of dengue. Nature- Letter Research 2013:5. doi: doi:10.1038/nature12060.http://dx.doi.org/10.1038/nature12060
2. Adalja AA, Sell TK, Bouri N, Franco C. Lessons learned during dengue outbreaks in the United States, 2001–2011. Emerg Infect Dis. 2012 http://dx.doi.org/10.3201/eid1804.110968 1.
3. Gubler DJ. Epidemic dengue/dengue hemorrhagic fever as a public health, social and economic problem in the 21st century. Trends Microbiol. 2002;10(2):100–3. doi: http://dx.doi.org/10.1016/S0966-842X(01)02288-0.
4. Hayden MH, Cavanaugh JL, Tittel C, Butterworth M, Haenchen S, Dickinson K, et al. Post outbreak review: Dengue preparedness and response in Key West, Florida. Am J Trop Med Hyg. 2015;93(2):397–400.http://www.ajtmh.org/content/93/2/397.abstract
5. Ooi E-E, Goh K-T, Gubler DJ. Dengue prevention and 35 years of vector control in Singapore. Emerg Infect Dis. 2006;12(6):887–93.http://www.ncbi.nlm.nih.gov/pmc/articles/PMC3373041/

## Supporting Information

S1 Table Historical and contemporary outbreaks of dengue in the 76 HICs (with year of classification as HICs). (DOCX)
