# Supplementary material for: Public Health Responses to and Challenges for the Control of Dengue Transmission in High-Income Countries: Four Case Studies
Source: PLoS Negl Trop Dis. 2016 Sep 19;10(9):e0004943. doi: 10.1371/journal.pntd.0004943 (PMC5028037; doi:10.1371/journal.pntd.0004943)
Supplement: S1 Table — (DOCX) [file pntd.0004943.s001.docx]

**S1 Table.** Historical and contemporary outbreaks of dengue in the 76 high income countries (with year of classification as high income countries) (A: Probable importation, no vector established; B: Only importation, no recent outbreak but presence of vector; C: recent local transmission, not epidemic, presence of vector; D: regular dengue outbreaks, epidemic, presence of vector; D: endemic, presence of vector)

| **Country** | **Year classification HICs** | **Current dengue status** | **Historical outbreaks** | **Vector present** |
| --- | --- | --- | --- | --- |
| **Andorra** | *1990* | Not epidemic (A) | No outbreak[^1^](#_ENREF_1) | No |
| **Antigua and Barbuda** | *2012* | Endemic (E) | Outbreaks in 1977-79, 1981, 2012. Few cases from 1994-2002, in 2008, 2010, 2011, 2012, 2013, 2014.^2,3^ | *Aedes aegypti*^4^ |
| **Aruba** | *1994* | Endemic (E) | Outbreak in 1984-1985, 1995, 1998-1999, 2009-14. Few cases in 1996, 2000, 2002, 2004, 2006, 2009.^2,3^ | *Ae. aegypti*^5^ |
| **Australia** | *1987* | Not endemic-Regular outbreaks (D) | Outbreaks in the early 1870s, 1879 (Townsville), 1885 (Charters Towers and Rockampton), 1894 (Thursday Island), 1895 (Townsville), 1897 (Queensland), 1898 (northern NSW). Outbreaks and deaths from 1899 to 1905 (Brisbane), 1909-1910, 1913-14, 1919-1923, 1926-1929 (northern Western Australia). Severe epidemic in Thursday Island in 1905. Epidemic in Brisbane in 1910-11. Dengue in the NT prior to 1914. Extensive epidemic in Queensland and some towns in north of NSW in 1916 and 1925-26.^6^ Large outbreaks in Queensland in 1941-43 and 1953-55. Reappearance of dengue in 1981.^7^ Regular locally-acquired cases since 1990 with large outbreaks in 1992-93 (Townsville, Charters Towers), 1996 (the Torres Strait), 1997-1999 (Cairns, Mossman and Port Douglas), 2003-2004 (Cairns, Townsville), 2008-2009 (Cairns, Townsville), 2010-2011 (Cairns, Townsville, Port Douglas, Mareeba, Cardwell).^8,9^ | *Ae. aegypti* in north Qld. *Aedes albopictus* in the Torres Strait.^10^ |
| **Austria** | *1987* | Only importation (A) | Outbreak in 1921.^11^ | No |
| **Bahamas, The** | *1987* | Not endemic- Regular but small outbreaks (C) | Outbreaks in 1977-79, 1982, 1989, 1998, 2003, 2011; Few cases in 1995, 2004, 2006, 2008, 2010, 2012, 2013, 2014.^2,3,12^ | *Ae. aegypti.*^12^ |
| **Bahrain** | *2001* | Not epidemic (A) | No outbreak.^1^ | *Ae. aegypti*.^13^ |
| **Barbados** | *2006* | Not endemic-Regular outbreaks (D) | Outbreaks in 1982, 1983, 1987, 1993, 1995-2005, 2010, 2011, 2012, 2013, 2014; few cases in 1981, 1988-1990, 1994, 2006.^3^ | *Ae. aegypti*. *Ae. albopictus* established in 1993.^14^ |
| **Belgium** | *1987* | Only importation (A) | No outbreak.^15^ | *Ae. albopictus* (not established)^16,17^ |
| **Bermuda** | *1987* | Not endemic-Regular but small outbreaks (C) | Outbreak in 1882, 1915, 1941.  Few cases in 1999, 2005, 2006, 2010, 2011, 2013.^2,3,18-20^ | *Ae. aegypti* eradicated in the mid-1960s, redetected in 1997 and *Ae. albopictus* first detected in 2000. Decline in *Ae. aegypti*, concurrent with the increase in *Ae. albopictus* populations.^21^ |
| **Brunei Darussalam** | *1987* | Endemic (E) | Outbreaks from 1992 to 2006, in 2010.^22,23^ | *Ae. aegypti*. *Ae. albopictus* detected in 2013.^24^ |
| **Canada** | *1987* | Only importation (A) | No outbreak.^25,26^ | No |
| **Cayman Islands** | *1993* | Not endemic-Regular but small outbreaks (C) | Few cases in 1998, 1999, 2002, 2003, 2005, 2007, 2008, 2010, 2011, 2012, 2013, 2014.^3,27^ | *Ae. aegypti* reported in 1938, eradicated in 1971 and reintroduced in 2002 after several reintroduction/successful elimination. *Ae. albopictus* established in 1997.^28^ |
| **Channel Islands** | *1987* | Not epidemic (A) | No outbreak.^1^ | No |
| **Chile** | *2012* | Outbreaks in Easter Island (C) | Outbreak in 2002 and 2009 in Easter Island.^29^  No reported indigenous cases in continental Chile.^30^ | *Ae. aegypti* eradicated in the 1950s in Chile, but detected in Easter Island in 2000.^31^ |
| **Croatia** | *2008* | Few outbreaks (C) | Outbreak in 2010.^32^ | *Ae. albopictus* first recorded in 2004.^33,34^ |
| **Curaçao** | *2010* | Endemic (E) | Outbreaks in 1968, 1977-79, 1993, 1995-1997, 2005, 2008, 2010-14. Few cases in 1998, 1999, 2000.^3,35-37^ | *Ae. aegypti* widely distributed.^2,38^ |
| **Cyprus** | *1988* | Not epidemic (B) | Outbreaks in 1861, 1888-1889, 1913, 1928.^39,40^ | *Ae. aegypti* reported.^17^ |
| **Czech Republic** | *2006* | Not epidemic (A) | No outbreak.^41^ | Imported *Ae. albopictus* in 2012.^42^ |
| **Denmark** | *1987* | Not epidemic (A) | No outbreak.^43^ | No |
| **Equatorial Guinea** | *2007* | Not epidemic (B) | No outbreak.^44^ | *Ae. albopictus* established in 2001 on the West African island of Bioko (Equatorial Guinea).^45^ |
| **Estonia** | *2006* | Not epidemic (A) | No outbreak.^1^ | No |
| **Faeroe Islands** | *1987* | Not epidemic (A) | No outbreak.^1^ | No |
| **Finland** | *1987* | Only importation (A) | No outbreak.^15,46^ | No |
| **France** | *1987* | Importations and few local transmissions (C) | Autochthonous cases in 2010 (two cases), 2013 (one case), 2014 (four cases).^47-50^  Outbreaks in Guyana in 1991, 2006, 2013, in Guadeloupe in 1994, in Martinique in 1995, 1997, 2001-2002, 2004-2005, 2005-2006, 2007-2008; in La Reunion in 1977-78, 2004, 20 autochthonous cases in 2012.^50,51^ | *Ae. albopictus* detected and established in 1999*.^16,34,52^*  *Ae. aegypti* reported but not established.^17^  *Ae. albopictus* in La Réunion |
| **French Polynesia** | *1990* | Epidemic and endemic periods (D) | Outbreak in 1882, 1943-44, 1964-65, 1969, 1971, 1975, 1979, 1985, 1988, 1989, 1990, 1996-97, 2001, 2002-2005, 2006, 2008, 2009 (large), 2010, 2011, 2013-2014.^53,54^ | *Ae. aegypti* reported in 1953 in Tahiti then spread in 1960.^55,56^ |
| **Germany** | *1987* | Only importation so far (A) | No outbreak^25,57-59^ | *Ae. albopictus* (not established)^16,17,34^ |
| **Greece** | *1996* | Only importation so far (B) | Large outbreak in 1881, 1927-1928.^60,61^. Outbreak in 1889, 1895-1897^62^, 1910, 1929-1933.^61,63^ | *Ae. albopictus* established in 2003.^16,34,64^  *Ae. aegypti* reported^17^ |
| **Greenland** | *1987* | Not epidemic (A) | No outbreak.^1^ | No |
| **Guam** | *1995* | Few importations (endemic^*^) (B) | Outbreaks in 1933-37, 1941-45.^65^  Few imported cases each year but no big outbreaks (M.O. Scroggs, Personal Communication, 2014) | *Ae. albopictus* reported in 1944 (introduced during the war).^66^  *Ae. aegypti* eliminated in 1945. *Ae. albopictus* dominant species in the 1960s.^65^ |
| **Hong Kong SAR, China** | *1987* | Few outbreaks (C) | Local transmission in 2002-2003.^67^ | *Ae. albopictus* only so far.^67^ |
| **Iceland** | *1987* | Not epidemic (A) | No outbreak.^1^ | No |
| **Ireland** | *1987* | Not epidemic (A) | No outbreak.^1^ | No |
| **Isle of Man** | *2002* | Not epidemic (A) | No outbreak.^1^ | No |
| **Israel** | *1987* | Only importation so far (B) | Outbreaks in 1889, 1912`^68^, 1927-1928, 1945.^40,69^ Since then no outbreak.^25,70^ | *Ae. albopictus* reported since 2002.^71^  *Ae. aegypti* eliminated in 1950.^72^; reoccurrence in 1975.^17, 73^ |
| **Italy** | *1987* | Only importation so far (B) | Outbreak in 1889-1890.^40^  Since then, no outbreak.^15,74^ | *Ae. albopictus* first recorded in 1990.^16,34,75^  *Ae. aegypti* reported.^17,72^ |
| **Japan** | *1987* | Importations and recent local transmissions (C) | Outbreaks in 1942-45 (importations from soldiers returning from the tropics).^76^  First locally acquired cases in 2014 since the 1940s.^77^ | *Ae. albopictus*.^16,78^  *Ae. aegypti* reported at International Airport in 2012. |
| **Korea, Rep.** | *2001* | Only importation so far (B) | No outbreak.^79^ | *Ae. albopictus*.^80^ |
| **Kuwait** | *1987* | Only importation (A) | No outbreak.^81^ | No |
| **Latvia** | *2012* | Only importation (A) | No outbreak.^15^ | No |
| **Liechtenstein** | *1994* | Not epidemic (A) | No outbreak.^1^ | No |
| **Lithuania** | *2012* | Only importation (A) | No outbreak.^15,82^ | No |
| **Luxembourg** | *1987* | Only importation (A) | No outbreak.^15^ | No |
| **Macao SAR, China** | *1994* | Importations and local transmissions (C) | Outbreak in 2001, 2014.^1^ | *Ae. albopictus.^83^* |
| **Malta** | *2002* | Not epidemic (B) | Outbreak in 1927. | *Ae. albopictus.*^84^ |
| **Monaco** | *1994* | Not epidemic (B) | No outbreak.^1^ | *Ae. albopictus*^16, 34^ |
| **Netherlands (The)** | *1987* | Only importation so far (A) | No outbreak.^85^ | *Ae. albopictus* recorded in 2005 (not established)^16,34,86, 87^ and *Ae. aegypti* in 2010 (not established).^17,88^ |
| **New Caledonia** | *1995* | Outbreaks but not endemic (D) | Outbreaks in 1884-85, 1989^89^, 1997, 1998-99, 2003-04, 2008-2009, 2012-2013.^90^ | *Ae. aegypti.*^91^ |
| **New Zealand** | *1987* | Only importation (A) | No outbreak.^92,93^ | Intercepted species: *Ae. aegypti* (not established) and *Ae. albopictus* (not established)^16,94^ |
| **Northern Mariana Islands** | *2007* | Not epidemic (B) | One case in 1998.^95^ | *Ae. aegypti* and *Ae. albopictus* reported in 1944. *Ae. aegypti* population declined after vector control program.^96,97^ |
| **Norway** | *1987* | Only importation (A) | No outbreak.^98^ | No |
| **Oman** | *2007* | Only importation (B) | No outbreak.^99^ | *Ae. aegypti* reported in 2008.^100^ |
| **Poland** | *2009* | Only importation (A) | No outbreak.^15,101^ | No |
| **Portugal** | *1994* | Outbreaks but not endemic (C) | Outbreak in 1889.^40^  Large outbreak in the Island of Madeira in 2012-2013 (> 2100 cases).^102-105^ | *Ae. aegypti* present in continental Portugal up to 1956. *Ae. aegypti* reported in the Island of Madeira since 2005.^106,107^ |
| **Puerto Rico** | *2002* | Endemic (E) | Outbreaks between 1827 and 1880, in 1945. Periodical outbreaks since 1963^108^ and four large outbreaks since 1990 (1994, 1998, 2007, 2010).^3,109^ 27,107 locally-acquired cases from 2010-14.^110^ | *Ae. aegypti*. |
| **Qatar** | *1987* | Not epidemic (A) | No outbreak.^1^ | No |
| **Russian Federation** | *2012* | Only importation (B) | No outbreak. | *Ae. albopictus* and *Ae. aegypti* reported.^111,112^ |
| **San Marino** | *2000* | Not epidemic (B) | No outbreak.^1^ | *Ae. albopictus*.^34,113^ |
| **Saudi Arabia** | *2004* | Outbreaks but not endemic (C) | Outbreaks in 1994-99^114^, 2004-2005.^115^ | *Ae. aegypti.*^116^ |
| **Singapore** | *1987* | Endemic (E) | Outbreaks from 1960-73, then few outbreaks from 1973-90. Large outbreaks 1990-2014.^117,118^ | *Ae. aegypti* (predominant), *Ae. albopictus* present.^119,120^ |
| **Sint Maarten (Dutch part)** | *2010* | Outbreaks but not endemic (C) | Outbreaks in 1977-79, 1981, 2010 | *Ae. aegypti^2^* |
| **Slovak Republic** | *2007* | Not epidemic (B) | No outbreak.^1^ | *Ae. albopictus^121^* |
| **Slovenia** | *1997* | Only importation (B) | No outbreak.^15^ | *Ae. albopictus*.^122^ |
| **Spain** | *1987* | Only importation (B) | Pandemic 1779-1884; Outbreak in 1863, 1867 (imported from the West Indies by troops).^40,123^ Since then, no outbreak.^15,124,125^ | *Ae. albopictus* established in 2004 (East Coast).^16,126,127^ |
| **St. Kitts and Nevis** | *2011* | Outbreaks but not endemic (D) | Outbreaks in 1977-1979^2^, 1995, 2013. Few cases in 1981, 1985, 1993-94, 1996-97, 1999-2004, 2006, 2008, 2009, 2010, 2011, 2012, 2014.^3^ | *Ae. aegypti* present in high densities |
| **St. Martin (French part)** | *2010* | Outbreaks but not endemic (D) | Outbreaks in 1977-1979^2^, 1981, 2005-2006, 2009, 2010, 2011, 2012, 2013, 2014.^3^ | *Ae. aegypti*. |
| **Sweden** | *1987* | Only importation (A) | No outbreak.^15^ | No |
| **Switzerland** | *1987* | Only importation (B) | No outbreak.^128, 129^ | *Ae. albopictus* established in 2003.^34^ |
| **Taiwan, China** | *1987* | Regular outbreaks (D) | Outbreaks in 1870, 1902, 1915, 1922, 1924, 1927, 1931, 1942-44, 1981 (large), 1987-88 (large), 1990-1996^130^, 1998, 2002 (large), 2006-2007 (large), 2009-2014 (imported and locally-acquired).^131^ | *Ae. albopictus.^16^*  *Ae. aegypti.*^132^ |
| **Trinidad and Tobago** | *2006* | Outbreaks but not endemic (D) | Outbreaks in 1952, 1953-54^133^, 1977-1979^2^, 1981-2008, 2010-14. Few cases in 2009.^3^ | *Ae. aegypti*. *Ae. albopictus* (not established).^16,126^,^127^ |
| **Turks and Caicos Islands** | *2009* | Few outbreaks (C) | Outbreak in 1977-79. Few cases in 2003, 2004, 2005, 2011, 2012, 2013, 2014.^3^ | *Ae. aegypti.*^2^ |
| **United Arab Emirates** | *1987* | Only importation (B) | No outbreak.^134^ | *Ae. aegypti*.^13^ |
| **United Kingdom** | *1987* | Only importation (A) | No outbreak.^15,135^ | No^136^ |
| **United States** | *1987* | Outbreaks but not endemic (C) | New Orleans: outbreaks in 1848-49, large outbreak in 1873, pandemic in 1879-1880.^18^ Texas: outbreaks in 1897, 1922, 1941, 2005. Florida: outbreaks in 1898-99, 1922, 1934, 2009-13.^137^ Hawaii: 2001-2002. No cases during 1946-1980, in continental United States. Locally-acquired dengue in 2009-2012.^3,138^ | *Ae. albopictus* detected in Texas in 1985. *Ae. albopictus* established in Alabama, Arkansas, Florida, Hawaii, Illinois, Indiana, Louisiana, Mississippi, Missouri, New Jersey, North Carolina, Ohio, South Carolina, Tennessee.^16,139^ *Ae. aegypti* in some states^137^ and recently reported in Los Angeles^140^ |
| **Uruguay** | *2012* | Only importation (B) | Autochthonous case in 1916. No outbreak.^30^ | *Ae. aegypti.^141^* |
| **Virgin Islands (U.S.)** | *1987* | Outbreaks but not endemic (C) | First recorded pandemic in Virgin Islands seaports in 1827.^18^ Outbreaks in 1977-79, 1981. Cases in 2007, 2013, 2014.^3^ | *Ae. Aegypti.^2,142^* |

1. Centers for Disease Control and Prevention. Dengue Map, http://www.healthmap.org/dengue/en/, accessed the 01/10/2014.

2. Knudsen AB. *Aedes aegypti* and dengue in the Caribbean. Mosquito News. 1983; **43**(3): 269-75.

3. Pan American Health Organization. Dengue regional information: Number of cases. Available at: http://www.paho.org/hq/index.php?option=com_topics&view=article&id=1&Itemid=40734. Accessed online the 24/09/2014. Regional Office for the Americas of the World Health Organization.

4. Verna TN, Munstermann LE. Morphological variants of *Aedes aegypti* collected from the Leeward Island of Antigua. J Am Mosq Control Assoc. 2011; **27**(3): 308-11.

5. Aruba Vector Control Department. Aruba Vector Control Department. Available online at: http://www.despa.aw/index.php?view=article&id=25%3Ayellow-fever-mosquito-control&format=pdf&option=com_content&Itemid=24; 2014.

6. Lumley GF. Dengue : Part I, Medical In: Australasian Medical Publishing Company Limited, editor. Dengue : Part I, Medical - Part II, Entomological. Glebe: School of Public Health and Tropical Medicine, University of Sydney; 1943. p. 171 p.

7. Kay BH, Barkerhudson P, Stallman ND, Wiemers MA, Marks EN, Holt PJ, et al. Dengue fever - reappearance in northern Queensland after 26 Years. Med J Aust. 1984; **140**(5): 264-8.

8. Ritchie S, Pyke A, Hall-Mendelin S, Day A, Mores C, Christofferson R, et al. An explosive epidemic of DENV-3 in Cairns, Australia. Plos One. 2013; **8**(7): e68137.

9. Viennet E, Ritchie S, Faddy H, Williams C, Harley D. Epidemiology of dengue in a high-income country: a case study in Queensland, Australia. Parasit Vectors. 2014; **7**(1): 379.

10. Ritchie SA, Moore P, Carruthers M, Williams C, Montgomery B, Foley P, et al. Discovery of a widespread infestation of *Aedes albopictus* in the Torres Strait, Australia. J Am Mosq Control Assoc. 2006; **22**(3): 358-65.

11. Ulmann E. The geographical distribution of dengue up to 1957. Hamburg, Welt-Seuchen-Atlas; 1961.

12. Pan American Health Organization, World Health Organization. Health in the Americas, Bahamas. Available at: http://www.paho.org/saludenlasamericas/index.php?id=22&option=com_content. Accessed online the 25/09/2014; Updated the 11 April 2013.

13. Rathor HR. The role of vectors in emerging and re-emerging diseases in the Eastern Mediterranean Region. Available at: http://repository.searo.who.int/handle/123456789/15829 Dengue Bulletin. 2000: 103-9.

14. Reiter P. *Aedes albopictus* and the world trade in used tires, 1988–1995: The shape of things to come? J Am Mosq Control Assoc 1998; **14**: 83-94.

15. European Centre for Disease Prevention and Control. Annual Epidemiological Report 2013. Reporting on 2011 surveillance data and 2012 epidemic intelligence data. Available online: http://www.ecdc.europa.eu/en/publications/publications/annual-epidemiological-report-2013.pdf. Stockholm: ECDC; 2013.

16. European Centre for Disease Prevention and Control. *Aedes albopictus.* Available at: http://www.ecdc.europa.eu/en/healthtopics/vectors/mosquitoes/Pages/aedes-albopictus.aspx. Accessed online the 24/09/2014. 2014.

17. European Centre for Disease Prevention and Control. *Aedes aegypti.* Available at: http://www.ecdc.europa.eu/en/healthtopics/vectors/mosquitoes/Pages/aedes-aegypti.aspx. Accessed online the 24/09/2014. 2014.

18. Ehrenkranz NJ, Ventura AK, Cuadrado RR, Pond WL, Porter JE. Pandemic dengue in Caribbean countries and the southern United States — Past, present and potential problems. N Engl J Med. 1971; **285**(26): 1460-9.

19. Foster FP. Last year's epidemic of dengue in Bermuda. New York Medical Journal. 1883; **28**(322-323).

20. Meagher ET. On dengue referring to an epidemic at Bermuda. Journal of the Royal Naval Medical Service. 1916; **2**: 188-90.

21. Kaplan L, Kendell D, Robertson D, Livdahl T, Khatchikian C. *Aedes aegypti* and *Aedes albopictus* in Bermuda: extinction, invasion, invasion and extinction. Biological Invasions. 2010; **12**(9): 3277-88.

22. Rahman ZH, Osman O, Muharram SH, Mabruk M. The prevalence of dengue virus in Brunei Darussalam during January-November 2010. Southeast Asian J Trop Med Public Health. 2013; **44**(4): 594-601.

23. Hisamonie Koh F, Misli H, Chong VH. Acute acalculous cholecystitis secondary to dengue fever Brunei Int Med J 2011; **7**(1): 45-9.

24. Idris FH, Usman A, Surendran SN, Ramasamy R. Detection of *Aedes albopictus* pre-imaginal stages in brackish water habitats in Brunei Darussalam. Journal of Vector Ecology. 2013; **38**(1): 197-9.

25. Schwartz E, Meltzer E, Mendelson M, Tooke A, Steiner F, Gautret P, et al. Detection on four continents of dengue fever cases related to an ongoing outbreak in Luanda, Angola, March to May 2013. Euro Surveill 2013; **18**(21): pii=20488. Available online: http://www.eurosurveillance.org/ViewArticle.aspx?ArticleId=.

26. World Health Organization. Human arboviral surveillance Weekly Epid Record. 1980; **55**(10): 76.

27. Lounibos LP, Escher RL, Lourenco-de-Oliveira R. Asymmetric evolution of photoperiodic diapause in temperate and tropical invasive populations of Aedes albopictus (Diptera: Culicidae) Ann Entomol Soc Am. 2003; **96**: 512–8.

28. Wheeler AW, Petrie WD. An overview of Aedes aegypti and Aedes albopictus control in the British Overseas Territory of the Cayman Islands. Euro Surveill. 2007; **12**(47): pii=3314. Available online: http://www.eurosurveillance.org/ViewArticle.aspx?ArticleId=.

29. Chowell G, Fuentes R, Olea A, Aguilera X, Nesse H, Hyman JM. The basic reproduction number R0 and effectiveness of reactive interventions during dengue epidemics: the 2002 dengue outbreak in Easter Island, Chile. Math Biosci Eng. 2013; **10**(5&6): 1455-74.

30. Zambrano B, San Martin JL. Epidemiology of dengue in Latin America. Journal of the Pediatric Infectious Diseases Society. 2014; **3**(3): 181-2.

31. Gobierno de Chile, Ministerio de Salud. El Vigia. *Aedes aegypti* en Isla de Pascua. Boletin de Vigilancia en Salud Publica de Chile. 2001; **13**.

32. Gjenero-Margan I, Aleraj B, Krajcar D, Lesnikar V, Klobucar A, Pem-Novosel I. Autochthonous dengue fever in Croatia, August-September 2010. Euro Surveill. 2011; **16**: pii=19805.

33. Klobucar A, Merdic E, Benic N, Baklaic Z, Krcmar S. First record of *Aedes albopictus* in Croatia. J Am Mosq Control Assoc. 2006; **22**(1): 147-8.

34. European Centre for Disease Prevention and Control. Technical report: Development of *Aedes albopictus* risk maps. Available online at http://www.ecdc.europa.eu/en/publications/publications/0905_ter_development_of_aedes_albopictus_risk_maps.pdf. Stockholm; 2009.

35. van der Sar A. An outbreak of dengue haemorrhagic fever on Curacao. Trop Geogr Med 1973; **25**: 119-29.

36. Weiland HT, Williams MC, Hull B. Serologic survey of dengue and other arboviruses in Curacao and Aruba, 1973. Bull Pan Am Health Organ. 1978; **12**(2): 134-42.

37. Limper M, Van de Weg C, Koraka P, Halabi Y, Gerstenbluth I, Boekhoudt J, et al. The 2008 dengue epidemic on Curacao: Correlation with climatological factors. International Journal of Infectious Diseases. 2010; **14**: E376-E7.

38. Pan American Health Organization, Pan American Sanitary Bureau, Organization ROotWH. Proceedings of the CAREC/PAHO Sub-Regional Dengue Meeting. June 1-3, 1999; Port of Spain Trinidad Division of Disease Prevention and Control,; June 1-3, 1999.

39. Brown AWA. Yellow fever, dengue and dengue haemorrhagic fever. In: Howe GM e, editor. A world geography of human diseases. NewYork: Academic Press; 1977. p. 271–317.

40. Ulmann E. Dengue epidemics in the Mediterranean area (1800–1950) and the distribution of its vector *Aedes aegypti*. In: Rodenwaldt E JH, eds, editor. Welt-Seuchen-Atlas. Hamburg: Falk-Verlag; 1956. p. 79–80.

41. Chalupa P, Kubek J, Hejlova A. Dengue fever in the Czech Republic. Brastisl Lek Listy. 2001; **102**(7): 322-5.

42. Šebesta O, Rudolf I, Betášová L, Peško J, Hubálek Z. An invasive mosquito species *Aedes albopictus* found in the Czech Republic, 2012. Euro Surveill 2012; **17**(43): pii=20301. Available online: http://www.eurosurveillance.org/ViewArticle.aspx?ArticleId=.

43. Vinner L, Domingo C, Ostby ACB, Rosenberg K, Fomsgaard A. Cases of travel-acquired dengue fever in Denmark 2001–2009. Clinical Microbiology and Infection. 2012; **18**(2): 171-6.

44. Amarasinghe A, Kuritsky JN, Letson GW, Margolis HS. Dengue virus infection in Africa. Available at http://dx.doi.org/10.3201/eid1708.101515. Emerg Infect Dis 2011 **17**(8).

45. Toto JC, Abaga S, Carnevale P, Simard F. First report of the oriental mosquito *Aedes albopictus* on the West African island of Bioko, Equatorial Guinea. Med Vet Entomol. 2003; **17**(3): 343-6.

46. Huhtamo E, Korhonen E, Vapalahti O. Imported dengue virus serotype 1 from Madeira to Finland 2012. Euro Surveill. 2013; **18**(8): 20405.

47. La Ruche G, Souares Y, Armengaud A, Peloux-Petiot F, Delaunay P, Despres P. First two autochthonous dengue virus infections in metropolitan France, September 2010. Euro Surveill. 2010; **15**: pii=19676.

48. Marchand E, Prat C, Jeannin C, Lafont E, Bergmann T, Flusin O, et al. Autochthonous case of dengue in France, October 2013. Euro Surveill. 2013; **18**(50): pii=20661. Available online: http://www.eurosurveillance.org/ViewArticle.aspx?ArticleId=.

49. Tarantola A, Quatresous I, Ledrans M, Lassel L, Krastinova E, Cordel H, et al. Dengue d’importation diagnostiquée en France métropolitaine, janvier 2001–décembre 2006. Medecine Et Maladies Infectieuses. 2009; **39**(1): 41-7.

50. Ledrans M, Dejour S. Cas importés de chikungunya et de dengue en France métropolitaine - Bilan de la surveillance à partir des données de laboratoire Avril 2005 – décembre 2007. Available at www.invs.sante.fr. Saint-Maurice; 2008.

51. Larrieu S, Dehecq JS, Balleydier E, Jaffar MC, Michault A, Vilain P, et al. Re-emergence of dengue in Réunion, France, January to April 2012. Euro Surveill. 2012; **17**(20): pii=20173. .

52. Schaffner F, Karch S. Premiere observation d’*Aedes albopictus* (Skuse, 1894) en France metropolitaine. Acad Sci Paris Sciences de la Vie. 2000; **323**: 373–5.

53. Pacific Public Health Surveillance Network, Réseau océanien de surveillance de la santé publique. Communicable Disease Monitoring. Routine Surveillance. Available online at http://www.spc.int/phs/PPHSN/Surveillance/Routine_reports.htm. Accessed on 01/10/2014. 2014.

54. Descloux E, Cao-Lormeau V-M, Roche C, De Lamballerie X. Dengue 1 diversity and microevolution, French Polynesia 2001–2006: Connection with epidemiology and clinics. PLoS Negl Trop Dis. 2009; **3**(8): e493.

55. Chungue E, Deparis X, Murgue B. Dengue in French Polynesia: major features, surveillance, molecular epidemiology and current situation. Dengue Bull. 1998; **22**: 74-87.

56. Paupy C, Vazeille-Falcoz M, Mousson L, Rodhain F, Failloux AB. *Aedes aegypti* in Tahiti and Moorea (French Polynesia): isoenzyme differentiation in the mosquito population according to human population density. Am J Trop Med Hyg. 2000; **62**(2): 217-24.

57. Schmidt-Chanasit J, Haditch M, Schoneberg I, Gunther S, Stark K, Frank C. Dengue virus infection in a traveller returning from Croatia to Germany. Euro Surveill. 2010; **15**: pii=19677.

58. Frank C, Höhle M, Stark K, Lawrence J. More reasons to dread rain on vacation? Dengue fever in 42 German and United Kingdom Madeira tourists during autumn 2012. . Euro Surveill 2013; **18**(14): pii=20446. Available online: http://www.eurosurveillance.org/ViewArticle.aspx?ArticleId=.

59. Schmidt-Chanasit J, Emmerich P, Tappe D, Günther S, Schmidt S, Wolff D, et al. Autochthonous dengue virus infection in Japan imported into Germany, September 2013. Euro Surveill. 2014; **19**(3): pii=20681. Available online: http://www.eurosurveillance.org/ViewArticle.aspx?ArticleId=.

60. Rosen L. Dengue in Greece in 1927 and 1928 and the pathogenesis of Dengue Hemorrhagic Fever: new data and a different conclusion. Am J Trop Med Hyg. 1986; **35**(3): 642-53.

61. Kuno G. Emergence of the severe syndrome and mortality associated with dengue and dengue-like illness: historical records (1890 to 1950) and their compatibility with current hypotheses on the shift of disease manifestation. Clin Microbiol Rev. 2009; **22**(2): 186-+.

62. Chastel C. Lessons from the Greek dengue epidemic of 1927-1928. Bulletin De L Academie Nationale De Medecine. 2009; **193**(2): 485-93.

63. Louis C. Daily newspaper view of dengue fever epidemic, Athens, Greece, 1927-1931. Emerg Infect Dis. 2012; **18**(1): 78-82.

64. Samanidou-Voyadjoglou A, Patsoula E, Spanakos G, Vakalis NC. Confirmation of *Aedes albopictus* (Skuse) (Diptera: Culicidae) in Greece. European Mosquito Bulletin. 2005; **19**: 10-1.

65. Ward RA. Mosquito fauna of Guam: case history of an introduced fauna. In: Laird ME, editor. Commerce and the spread of pests and disease vectors New York: Praeger Scientific; 1984. p. 354.

66. Hu SMK. Mosquito survey of Guam. Mosquito News. 1953; **13**(2): 123-5.

67. Chuang VWM, Wong TY, Leung YH, Ma ESK, Law YL, Tsang OTY, et al. Review of dengue fever cases in Hong Kong during 1998 to 2005. Hong Kong Med J. 2008; **14**(3): 170-7.

68. Masterman EWG. Notes on some tropical diseases of Palestine. J Hyg. 1913; **13**: 49–62.

69. Hitti JK, Khairallah AA. A report on the recent epidemic of dengue in Beirut, Lebanon, and some of its complications. J Palestine Arab Med Ass. 1946; **1**: 150–53.

70. Schwartz E. Study of dengue fever among Israeli travellers to Thailand. Dengue Bulletin. 2002; **26**: 162-7.

71. Leshem E, Bin H, Shalom U, Perkin M, Schwartz E. Risk for emergence of dengue and chikungunya virus in Israel. Emerg Infect Dis. 2012; **18**(2): 345-7.

72. Holstein M. Dynamics of *Aedes aegypti* distribution, density and seasonal prevalence in the Mediterranean area. Bulletin of the World Health Organization. 1967; **36**: 541-3.

73. Pener-Salomon H, Vardi A. Reoccurence of *Aedes aegypti* (Insecta: Diptera: Culicidae) in Israel. Israel Journal of Zoology. 1975; **24**: 193.

74. Napoli C, Salcuni P, Pompa MG, Declich S, Rizzo C. Estimated imported infections of chickungunya and dengue in Italy, 2008 to 2011. J Travel Med. 2012: 1195-982.

75. Sabatini A, Raineri V, Trovato G, Coluzzi M. *Aedes albopictus* in Italy and possible diffusion of the species into the Mediterranean area. Parassitologia. 1990; **32**(3): 301-4.

76. Hotta S. Dengue epidemics in Japan, 1942-1945. J Trop Med Hyg. 1953; **56**(4): 83.

77. Dolan N. Dengue in Japan. Lancet Infect Dis. 2014; **14**(10): 927.

78. Kobayashi M, Komagata O, Yonejima M, Maekawa Y, Hirabayashi K, Hayashi T, et al. Retrospective search for dengue vector mosquito Aedes albopictus in areas visited by a German traveler who contracted dengue in Japan. International Journal of Infectious Diseases. 2014; **26**: 135-7.

79. Park J-H, Lee D-W. Dengue fever in South Korea, 2006–2010. Available online at http://dx.doi.org/10.3201/eid1809.111811 Emerg Infect Dis. 2012 **18**(9): 1525-7.

80. Tanaka K, Mizusawa K, Saugstad ES. A revision of the adult and larval mosquitoes of Japan (including the Ryukyu Archipelago and the Ogasawara Islands) and Korea (Diptera: Culicidae) Contrib Amer Ent Inst 1979; **16**: 1-987.

81. Mustafa AS, Elbishbishi EA, Grover S, Pacsa AS, Al-Enezi AA, Chaturvedi UC. A study of dengue imported to Kuwait during 1997-1999. Acta Virologica. 2001; **45** (2): 125-8.

82. ULAC. Case of imported dengue fever was reported in Lithuania. Available at: http://www.ulac.lt/en/naujienos/press-releases/case-of-imported-dengue-fever-was-reported-in-lithuania. 2013.

83. Almeida APG, Baptista SSSG, Sousa CAGCC, Novo MTLM, Ramos HC, Panella NA, et al. Bioecology and vectorial capacity of *Aedes albopictus* (Diptera: Culicidae) in Macao, China, in relation to dengue virus transmission. J Med Entomol. 2005; **42**(3): 419-28.

84. Gatt P, Deeming JC, Schaffner F. First record of *Aedes* (Stegomyia) *albopictus* (Skuse) (Diptera: Culicidae) in Malta. European Mosquito Bulletin. 2009; **27**: 56-64.

85. Bakker RC, Veenstra J, Dingemans-Dumas AM, Wetsteyn JCFM, Kager PA. Imported Dengue in The Netherlands. J Travel Med. 1996; **3**(4): 204-8.

86. Scholte E, Jacobs F, Linton Y, Dijkstra E, Fransen J, Takken W. First record of *Aedes* (Stegomyia) *albopictus* in the Netherlands. Eur Mosquito Bull. 2007; **22**: 5 - 9.

87. Scholte E-J, Den Hartog W, Dik M, Schoelitsz B, Brooks M, Schaffner F. Introduction and control of three invasive mosquito species in the Netherlands, July-October 2010. Euro Surveill. 2010; **15**: pii=19710.

88. Brown J, Scholte E-J, Dik M, Den Hartog W, Beeuwkes J, Powell J. *Aedes aegypti* mosquitoes imported into the Netherlands, 2010. Emerg Infect Dis. 2011; **17**: 2335 - 7.

89. Laille M, Huerre M, Flye Saint Marie F. An epidemic of dengue in 1989 in New Caledonia. Bull Soc Pathol Exot. 1990; **83**(5): 591-5.

90. Dupont-Rouzeyrol M, Aubry M, O'Connor O, Roche C, Gourinat A-C, Guigon A, et al. Epidemiological and molecular features of dengue virus type-1 in New Caledonia, South Pacific, 2001-2013. Virology Journal. 2014; **11**(1): 61.

91. Perry WJ. The dengue vector on New Caledonia, the new hebrides, and the Solomon Islands. Am J Trop Med Hyg. 1948; **s1-28**(2): 253-9.

92. Khan R, Lopez L, Baker M. Rise in imported dengue fever in New Zealand. New Zealand Public Health Report. 2001; **8**(11): 81-8.

93. Centre for Diseases Control. DengueMap. Available online at: http://www.healthmap.org/dengue/en/.

94. Derraik JGB. Exotic mosquitoes in New Zealand: a review of species intercepted, their pathways and ports of entry. Aust N Z J Public Health. 2004; **28**(5): 433-44.

95. World Health Organization. Dengue in the WHO Western Pacific Region. Weekly Epid Record. 1998; **36**: 273-7.

96. Nowell WR. Mosquito survey, Island of Rota (Mariana Islands). San Fransisco; 1976.

97. Savage HM, Mitchell CJ, Roppul M, Castro LT, Kepple RL, Flood SP. Mosquito faunal survey of Saipan, Mariana Islands (Diptera: Culicidae): Taxonomy and larval ecology. Mosquito Systematics. 1993; **25**(1): 17-24.

98. Vainio K, Noraas S, Holmberg M, Fremstad H, Wahlstrøm M, Ånestad G, et al. Fatal and mild primary dengue virus infections imported to Norway from Africa and south-east Asia, 2008-2010. Euro Surveill. 2010; **15**(38): pii=19666. Available online: http://www.eurosurveillance.org/ViewArticle.aspx?ArticleId=.

99. Al Awaidy ST, Al Obeidani I, Bawikar S, Al Mahrouqi S, Al Busaidy SS, Al Baqlani S, et al. Dengue epidemiological trend in Oman: a 13-year national surveillance and strategic proposition of imported cases. Tropical Doctor. 2014; **44**(4): 190-5.

100. Sultanate of Oman, Ministry of HEALTH. Emerging infectious diseases- Part 1; July-August 2010.

101. Goljan J, Myjak P, Nahorski W, Kubica-Biernat B, Felczak-Korzybska I, Kowalczyk D, et al. Dengue antibodies in Polish travellers returning from the tropics. Evaluation of serological tests. Int Marit Health. 2010; **61**(1): 36-40.

102. Sousa C, Clairouin M, Seixas G, Viveiros B, Novo M, Silva A. Ongoing outbreak of dengue type 1 in the autonomous region of Madeira (Portugal): preliminary report. Euro Surveill. 2012; **17**: pii=20333.

103. European Centre for Disease Prevention and Control. Epidemiological update: Outbreak of dengue in Madeira, Portugal. Available onlie at http://www.ecdc.europa.eu/en/press/news/_layouts/forms/News_DispForm.aspx?List=8db7286c-fe2d-476c-9133-18ff4cb1b568&ID=23 Epidemiological update; 2013.

104. Tomasello D, Schlagenhauf P. Chikungunya and dengue autochthonous cases in Europe, 2007-2012. Travel Med Infect Dis. 2013; **11**(5): 274-84.

105. Wilder-Smith A, Quam M, Sessions O, Rocklov J, Liu-Helmersson J, Franco L, et al. The 2012 dengue outbreak in Madeira: exploring the origins. Euro Surveill. 2014; **19**(8): pii=20718. Available online: http://www.eurosurveillance.org/ViewArticle.aspx?ArticleId=

106. Alves MJ, Fernandes PL, Amaro F, Osório H, Luz T, Parreira P, et al. Clinical presentation and laboratory findings for the first autochthonous cases of dengue fever in Madeira island, Portugal, October 2012. Euro Surveill. 2013; **18**(6): pii=20398. Available from: http://www.eurosurveillance.org/ViewArticle.aspx?ArticleId=.

107. Almeida A, Goncalves Y, Novo M, Sousa C, Melim M, Gracio A. Vector monitoring of Aedes aegypti in the Autonomous Region of Madeira, Portugal. Euro Surveill. 2007; **12**: pii=3311.

108. Morales H, Madera JE, Ramirez-Ronda CH, Bermudez R, Gonzales V, H. R. Follow-up on dengue-Puerto Rico. Morbidity and Mortality Weekly Reports. 1976; **25**(7).

109. Centers for Disease Control and Prevention. Dengue in Puerto Rico. Available online at : http://www.cdc.gov/dengue/about/inPuerto.html. Accessed the 23/09/2014. Dengue Homepage: USA Government; 2014.

110. U.S. Department of the Interior, U.S. Geological Survey. Dengue fever (locally acquired) Human. Available online at http://diseasemaps.usgs.gov/del_us_human.html. CDC,; 2014.

111. Ganushkina LA, Dremova VP. *Aedes aegypti* L. and *Aedes albopictus* Skuse mosquitoes are a new biological threat to the south of Russia. Med Parazitol. 2012; **3**: 49-55.

112. Ganushkina LA, Tanygina E, Bezzhonova OV, Sergiev VP. Detection of *Aedes* (Stegomyia) *albopictus* skus. Mosquitoes in the Russian Federation. Med Parazitol. 2012; **1**: 3-4.

113. Medlock JM, Hansford KM, Schaffner F, Versteirt V, Hendrickx G, Zeller H, et al. A review of the invasive mosquitoes in Europe: ecology, public health risks, and control options. Vector Borne Zoonotic Dis. 2012; **12**(6): 435-47.

114. Fakeeh M, Zaki AM. Virologic and serologic surveillance for dengue fever in Jeddah, Saudi Arabia, 1994-1999. Am J Trop Med Hyg. 2001; **65**(6): 764-7.

115. Khan NA, Azhar EI, Ei-Fiky S, Madani HH, Abuljadial MA, Ashshi AA, et al. Clinical profile and outcome of hospitalized patients during first outbreak of dengue in Makkah, Saudi Arabia. Acta Trop. 2008; **105**(1): 39-44.

116. Alikhan M, Ghamdi KA, Mahyoub JA. *Aedes* mosquito species in western Saudi Arabia. Journal of Insect Science. 2014; **14**(69): 1-7.

117. Pinheiro F, Corber S. Global situation of dengue and dengue haemorrhagic fever, and its emergence in the Americas. World Health Stat Q. 1997; **50**(3-4): 161 - 9.

118. Ooi E-E, Goh K-T, Gubler DJ. Dengue prevention and 35 years of vector control in Singapore. Emerg Infect Dis. 2006; **12**(6): 887-93.

119. Chan YC, Chan KLB, Ho C. *Aedes aegypti* (L.) and *Aedes albopictus* (Skuse) in Singapore City. 1. Distribution and density. Bull World Health Organ. 1971; **44**: 617-27.

120. Lee C, Vythilingam I, Chong C-S, Abdul Razak MA, Tan C-H, Liew C, et al. Gravitraps for management of dengue clusters in Singapore. Am J Trop Med Hyg. 2013; **88**(5): 888-92.

121. Bocková E, Kočišová A, Letková V. First record of *Aedes albopictus* in Slovakia. Acta Parasitologica. 2013; **58**(4): 603-6.

122. Seyler T, Grandesso F, Le Strat Y, Tarantola A, Depoortere E. Assessing the risk of importing dengue and chikungunya viruses to the European Union. Epidemics. 2009; **1**(3): 175-84.

123. Armstrong C. Dengue Fever. Public Health Reports (1896-1970). 1923; **38**(31): 1750-84.

124. Ramos Geldres TT, Garcia Lopez-Hortelano M, Baquero-Artigao F, Montero Vega D, Lopez Quintana B, Mellado Pena MJ. Imported dengue: An emerging arbovirosis in Spain. An Pediatr. 2014; **28**(14): 00218-5.

125. Pinazo Delgado MJ, Muñoz-Gutierrez J, Betica-Radic L, Maretic T, Zekan S, Avšič-Županc T, et al. Imported dengue hemorrhagic fever, Europe. Available from http://wwwnc.cdc.gov/eid/article/14/8/08-0032. Emerg Infect Dis. 2008 **14**(8).

126. Alarcón-Elbal PM, Estrella SD, Arrondo IR, Collantes F, Iniesta JAD, Morales-Bueno J, et al. Updated distribution of *Aedes albopictus* (Diptera: Culicidae) in Spain: new findings in the mainland Spanish Levante, 2013. Memorias Do Instituto Oswaldo Cruz. 2014; **109**: 782-6.

127. Ministerio de Sanidad SSEI. Evaluación del riesgo de introducción y circulación del virus de dengue en España. Available online at: file:///E:/a%20articles/Biblio%20dengue/2013/2013_evaluacion%20del%20riesco%20de%20introduccion%20y%20circilacion%20del%20virus%20de%20dengue%20en%20%20Espana.pdf; 2013.

128. Settah SG, Vernazza PL, Morant R, Schultze D. Imported dengue fever in Switzerland--serological evidence for a hitherto unexpectedly high prevalence. Schweiz Med Wochenschr. 1995; **125**(36): 1673-8.

129. ProMED-mail. Dengue hem. fever, imported - Switzerland from India. 199612031995 Available online from http://wwwpromedmailorg/ Accessed 07/10/2014; ProMED-mail : 1996; 23 Mar.

130. Wu YC, Lien JC, Chen HY. Recent outbreak of dengue in Taiwan. TropMed. 1993; **35**(4): 201-7.

131. King C-C, Wu Y-C, Chao D-Y, Lin T-H, Chow L, Wang H-T, et al. Major epidemics of dengue in Taiwan in 1981-2000: Related to intensive virus activities in Asia. Dengue Bulletin. 2000; **24**: 1-10.

132. Wu H-H, Wang C-Y, Teng H-J, Lin C, Lu L-C, Jian S-W, et al. A dengue vector surveillance by human population-stratified ovitrap survey for *Aedes* (Diptera: Culicidae) adult and egg collections in high dengue-risk areas of Taiwan. J Med Entomol. 2013; **50**(2): 261-9.

133. Downs WG. Human infections with arboviruses in Trinidad, W. I. Am J Trop Med Hyg. 1964; **13**: 377-82.

134. Terzian C, Sheek-Hussein M, Nagelkerke N. The first confirmed case of imported dengue fever in the United Arab Emirates: Clinical, public health, and epidemiological aspects. Emirates Medical Journal. 2007; **25**(1): 61-4.

135. Lawn SD, Tilley R, Lloyd G, Finlayson C, Tolley H, Newman P, et al. Dengue hemorrhagic fever with fulminant hepatic failure in an immigrant returning to Bangladesh. Clin Infect Dis. 2003; **37**(1): e1-e4.

136. Burgess NRH. *Aedes albopictus*: a potential problem in the United Kingdom. Parassitologia. 1995; **37**(2-3): 121-2.

137. Beaumier C, Garcia MN, Murray KO. The history of dengue in the United States and its recent emergence. Current Tropical Medicine Reports. 2014; **1**: 32-5.

138. Centers for Disease Control and Prevention. Locally acquired dengue--Key West, Florida, 2009-2010. MMWR Morb Mortal Wkly Rep. 2010; **59**(19): 577-81.

139. Moore CG, Mitchell CJ. *Aedes albopictus* in the United States: ten-year presence and public health implications. Emerg Infect Dis. 1997; **3**(3): 329-4.

140. ProMED-mail. Invasive mosquito - USA (03): California. International Society for Infectious Diseases; Wed 15 Oct 2014.

141. Lourenco-de-Oliveira R, Rua A, Vezzani D, Willat G, Vazeille M, Mousson L, et al. *Aedes aegypti* from temperate regions of South America are highly competent to transmit dengue virus. BMC Infectious Diseases. 2013; **13**(1): 610.

142. Invasive Species Specialist Group. Global invasive species database. *Aedes aegypti*. Available from: http://www.issg.org/database/species/distribution.asp?si=1003&fr=1&sts=sss&lang=EN [Accessed 24/09/2014]. 2005.
